# A human liver organoid platform for hepatotoxicity assessment: evaluation using reference compounds

**DOI:** 10.3389/ftox.2026.1805474

**Published:** 2026-06-15

**Authors:** Min Jeong Kim, Seo Yoon Choi, Su Min Youn, Tae Sung Kim, Il Ung Oh, Sang Aeh Park, Jae Ho Oh, Myung Jin Son, Jin Hee Lee

**Affiliations:** 1 Division of Toxicological Research, National Institute of Food and Drug Safety Evaluation, Ministry of Food and Drug Safety, Cheongju, Republic of Korea; 2 Stem Cell Convergence Research Center, Korea Research Institute of Bioscience and Biotechnology (KRIBB), Daejeon, Republic of Korea; 3 Department of Advanced Bioconvergence, Korea University of Science & Technology (UST), Daejeon, Republic of Korea

**Keywords:** 3D culture, alternative to animal testing, liver organoid, liver toxicity, toxicity assessment

## Abstract

**Background:**

Recent advances in organoid technology have enabled the development of human-relevant models. However, their application in toxicity assessments remains limited. Our previous study employed a liver organoid platform to evaluate drug toxicity and subsequently suggested its utility as a model for toxicity assessment. Building on this, we aimed to assess the exploratory response of the liver organoid model using a panel of selected reference compounds, including substances classified according to the United Nations Globally Harmonized System acute toxicity criteria.

**Methods:**

Human liver organoids derived from induced pluripotent stem cells were exposed to a panel of reference compounds, and toxicity responses were evaluated using multiple endpoints, including cell viability assays, functional biomarker measurements, imaging, and transcriptomic analysis.

**Results:**

Exposure to the reference compounds resulted in compound-specific changes across multiple endpoints, including cell viability, hepatic functional markers such as albumin secretion, and transcriptomic responses. Transcriptomic analysis further revealed compound-specific changes in gene expression associated with hepatotoxic responses, supporting the observed functional alterations. These findings suggest that liver organoids may capture compound-specific and multi-dimensional toxicity responses, supporting their potential as an *in vitro* platform for integrative toxicity assessment. However, this study was conducted using a limited set of reference compounds and did not include direct comparisons with established *in vitro* or *in vivo* models. In addition, functional metabolic capacity, including CYP activity, was not assessed.

**Conclusion:**

Therefore, the present results should be interpreted as an exploratory evaluation of platform performance rather than evidence of predictive capability. Future studies incorporating greater chemical diversity and functional validation are required to assess the broader applicability of this model.

## 1 Introduction

In recent decades, substantial efforts have been directed towards developing reliable *in vitro* models for hepatotoxicity assessment during drug development (Shinozawa et al., 2021; Vorrink et al., 2018; Zhang et al., 2023). However, conventional models, including two-dimensional (2D) hepatocyte cultures and immortalized cell lines, often demonstrate limited physiological relevance, highlighting the need for more advanced human-relevant systems. Conventionally, toxicity assessments are performed using *in vitro* assays and animal models, whereas direct testing in humans remains limited because of ethical and practical constraints. However, conventional 2D cell models, which are advantageous in terms of reproducibility and standardization, fail to fully reflect the complex cellular interactions observed *in vivo* and have a limited capacity to capture precise effects on specific cell subpopulations (Krewski et al., 2010; Parasuraman, 2011). In this context, the use of well-characterized reference compounds has become an important approach for evaluating emerging *in vitro* platforms. By examining responses to selected hepatotoxicants and non-broadly toxic controls, such studies may provide initial insights into compound-associated responses in emerging *in vitro* models. These efforts represent a foundational step toward the further development and refinement of *in vitro* hepatotoxicity platforms.

Primary hepatocytes, directly isolated from human or animal liver tissue, are considered the gold-standard *in vitro* model because of their preserved liver-specific functions. Although physiologically similar to human liver cells, primary hepatocytes are limited by their short lifespans, restricted availability, and donor-to-donor variability. Therefore, animal models and animal-derived cells have been widely used to predict hepatotoxicity. However, these do not fully recapitulate human liver physiology. To address these limitations, three-dimensional (3D) cell culture–based organoid models have recently attracted considerable attention (Fatehullah et al., 2016; Kim et al., 2020; Nguyen et al., 2016). Organoids are miniaturized tissue-like structures derived from induced pluripotent or adult stem cells that self-organize in a 3D environment to mimic the architecture and functionality of actual organs. Organoids can partially recapitulate certain structural and functional features, which conventional models fail to achieve. They are considered intermediate models with the potential to bridge the gap between animal experiments and clinical data in drug development studies. Organoids from various organs, such as the liver, lungs, and kidneys, have been developed and are increasingly used in precision medicine, toxicology, and drug screening (Cho et al., 2022; Kim et al., 2018; Lee et al., 2024; Shao et al., 2025).

Although the use of liver organoids to evaluate chemical toxicity, particularly of non-pharmaceutical compounds, is limited, recent studies have increasingly used organoid-based models for broader toxicological applications. This growing trend reflects the expanding recognition of organoids as physiologically relevant platforms for chemical safety assessments (Bridgeman et al., 2025). Our previous study showed that liver organoids cultured in hepatic (HM) and differentiation (DM) medium comprised several liver-related cell types, including immature and mature hepatocyte-like cells, cholangiocytes, hepatic stellate cells, proliferative cells, and bipotent progenitors (Kim et al., 2023). These organoids not only retained key hepatic functions but also successfully distinguished between hepatotoxic and non-hepatotoxic substances, supporting their utility in evaluating compound-induced responses (Choi et al., 2024; Mun et al., 2019; Shin et al., 2025).

Therefore, the present study aimed to explore the utility of liver organoid models as *in vitro* platforms for evaluating hepatotoxic responses using selected reference compounds. Ten chemicals categorized according to the United Nations Globally Harmonized System were assessed across multiple endpoints. This approach extends the evaluation of organoid responses to a defined set of reference compounds, provides an initial multi-endpoint characterization of the model, and may help to support the potential utility of liver organoids as an emerging *in vitro* platform for chemical toxicity studies.

## 2 Materials and methods

### 2.1 Organoid culture and differentiation

Organoids were generated from induced pluripotent stem cells derived from normal human skin fibroblasts (CRL-2097) purchased from the American Type Culture Collection (Manassas, VA, United States). The organoid generation protocol has been described in detail in a previous study (Mun et al., 2023). For organoid culture, mature hepatocyte organoids were embedded in Matrigel (Catalog No. 354234; Corning Life Sciences, Tewksbury, MA, United States) with HM containing 10 μM Y-27632 (Catalog No. 1254; Tocris, Bristol, United Kingdom) for 3 days to improve viability. After the 3D liver organoids were formed in 3–5 days, the cell medium was changed to HM every 2–3 days. Every week, the expanded organoids were passaged at a ratio of 1:3–1:5. Organoids were split into small fragments using a tissue chopper (Catalog No. TC752; McIlwain; Ted Pella Inc., Redding, CA, United States) and resuspended in Matrigel. For further differentiation, liver organoids were incubated in expansion medium (EM) for 3 days, followed by DM for 8 days. Tables 1–3 detail the composition of the cell media.

**TABLE 1:** Composition of hepatic medium.

Reagent	Company	Catalog number	Concentration
Advanced DMEM/F12	Thermo Fisher scientific^1^	12634028	1×
Penicillin streptomycin	Thermo Fisher scientific^1^	15140122	1%
GlutaMAX	Thermo Fisher scientific^1^	35050079	1%
HEPES (1 M)	Thermo Fisher scientific^1^	15630080	10 mM
N2 supplement (100×)	Thermo Fisher scientific^1^	17502048	1×
N-Acetylcysteine	Sigma–Aldrich^2^	A9165	1 mM
[Leu15]-Gastrin I human	Sigma–Aldrich^2^	G9145	10 nM
Recombinant human EGF	PeproTech^3^	AF-100-15	50 ng/mL
Recombinant human HGF	PeproTech^3^	100-39	25 ng/mL
B27 supplement without Vit A	Thermo Fisher scientific^1^	12587010	1×
A83-01	Tocris bioscience^4^	2939	5 μM
Nicotinamide	Sigma–Aldrich^2^	N0636	10 mM
Forskolin	Sigma–Aldrich^2^	F3917	10 μM
Recombinant human FGF-basic	PeproTech^3^	100-18B	10 ng/mL
Oncostatin M	R&D systems^5^	295-OM	10 ng/mL
ITS (100×)	Thermo Fisher scientific^1^	41400045	5 μg/mL (1×)
Dexamethasone	Sigma–Aldrich^2^	D4902	100 nM

1 Waltham, MA, United States.

2 St. louis, MO, United States.

3 Cranbury, NJ, United States.

4 Bristol, United Kingdom.

5 Minneapolis, MN, United States

**TABLE 2:** Composition of expansion medium.

Reagent	Company	Catalog number	Concentration
Advanced DMEM/F12	Thermo Fisher scientific	12634028	1×
Penicillin streptomycin	Thermo Fisher scientific	15140122	1%
GlutaMAX	Thermo Fisher scientific	35050079	1%
HEPES (1 M)	Thermo Fisher scientific	15630080	10 mM
N2 supplement (100×)	Thermo Fisher scientific	17502048	1×
N-Acetylcysteine	Sigma–Aldrich	A9165	1 mM
[Leu15]-Gastrin I human	Sigma–Aldrich	G9145	10 nM
Recombinant human EGF	PeproTech	AF-100-15	50 ng/mL
Recombinant human HGF	PeproTech	100-39	25 ng/mL
B27 supplement without Vit A	Thermo Fisher scientific	12587010	1×
A83-01	Tocris bioscience	2939	5 μM
Nicotinamide	Sigma–Aldrich	N0636	10 mM
Forskolin	Sigma–Aldrich	F3917	10 μM
Recombinant human R-spondin	R&D systems	4645-RS-025	1 μg/mL
Recombinant human FGF10	PeproTech	100-26	100 ng/mL
Recombinant human BMP7	PeproTech	120-03P	25 ng/mL

**TABLE 3:** Composition of differentiation medium.

Reagent	Company	Catalog number	Concentration
Advanced DMEM/F12	Thermo Fisher scientific	12634028	1×
Penicillin streptomycin	Thermo Fisher scientific	15140122	1%
GlutaMAX	Thermo Fisher scientific	35050079	1%
HEPES (1 M)	Thermo Fisher scientific	15630080	10 mM
N2 supplement (100×)	Thermo Fisher scientific	17502048	1×
N-Acetylcysteine	Sigma–Aldrich	A9165	1 mM
[Leu15]-Gastrin I human	Sigma–Aldrich	G9145	10 nM
Recombinant human EGF	PeproTech	AF-100-15	50 ng/mL
Recombinant human HGF	PeproTech	100-39	25 ng/mL
B27 supplement with Vit A	Thermo Fisher scientific	17504044	1×
A83-01	Tocris bioscience	2939	0.5 μM
Dexamethasone	Sigma–Aldrich	D4902	3 μM
DAPT	Sigma–Aldrich	D5942	10 μM
Recombinant human BMP7	PeproTech	120-03P	25 ng/mL
Recombinant human FGF19	PeproTech	100-32	100 ng/mL

### 2.2 Solubility test

To validate the applicability of liver organoids for chemical-induced hepatotoxic testing, 10 representative chemicals—5 hepatotoxic [α-naphthyl isothiocyanate (ANIT), quizalofop-p-ethyl, carbonyl cyanide 4-(trifluoromethoxy) phenylhydrazone (FCCP), rotenone, and oligomycin) and 5 non-hepatotoxic (sodium acetate, D-mannitol, glucose, sorbitol, and glycine)—were selected. Hepatotoxicity was classified according to the Globally Harmonized System (Table 4). Before chemical treatment, solubility tests were conducted for all compounds (Supplementary Figure S1). Briefly, hydrophilic compounds were initially dissolved in distilled water (DW), while hydrophobic compounds were dissolved in dimethyl sulfoxide (DMSO) at their maximum intended concentrations, up to 1 M. When crystals were detected, the stock solution was diluted 10-fold until the crystals were no longer visible. For DMSO-solubilized compounds, the final concentration of DMSO in the culture medium did not exceed 1% (v/v). Vehicle control groups containing an equivalent concentration of DMSO were included to account for potential solvent-related effects. Microscopic images of each chemical dissolved in the cell culture medium were obtained to visually confirm complete solubility. Representative images are shown in Supplementary Figure S2. The stock solution was diluted 100-fold in the cell culture medium to establish the highest treatment concentration. The subsequent 10-fold dilutions were performed to obtain six concentrations for toxicity evaluation. The solubility test results for the 10 chemical compounds are presented in Supplementary Table S1.

**TABLE 4:** Globally Harmonized System classification of the 10 chemical compounds.

Substance name	CAS no.	Hazard class	Hazard statement	Signal word	Pictogram
ANIT	551-06-4	Acute toxicity (Oral, Dermal)	H301: Toxic if swallowed H311: Toxic in contact with skin	Danger	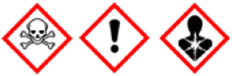
FCCP	370-86-5	Acute toxicity (Oral)	H301: Toxic if swallowed	Danger	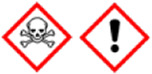
Oligomycin	1404-19-9	Acute toxicity (Oral)	H301: Toxic if swallowed	Danger	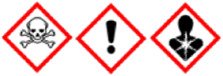
Quizalofop-p-ethyl	100646-51-3	Acute toxicity (Oral)	H302: Harmful if swallowed	Danger	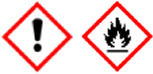
Rotenone	83-79-4	Acute toxicity (Oral)	H301: Toxic if swallowed	Danger	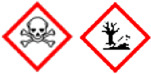
Sodium acetate	127-09-3	Not classified			
D-mannitol	69-65-8				
Glucose	50-99-7				
Sorbitol	50-70-4				
Glycine	56-40-6				

### 2.3 Holotomography

For holotomography, the HM and day 8 DM organoids were collected and transferred to a TomoDish (Catalog No. 901002-02; Tomocube Inc., Daejeon, Republic of Korea). Holotomography imaging was performed using an HT-X1™ Plus system (Tomocube Inc.), a low-coherence holotomography system that minimizes coherent noise. The HT-X1™ Plus system uses a light-emitting diode at a wavelength of 660 nm and is equipped with a ×40 motorized objective lens with a numerical aperture of 0.95. In addition, the system uses a digital micromirror device coupled with a condenser lens (NA = 0.72, working distance = 30 mm) to modulate the illumination patterns at the pupil plane.

### 2.4 Albumin Enzyme-Linked Immunosorbent Assay

To measure albumin (ALB) secretion, culture supernatants were collected 24 h after medium replacement. ALB was quantified using a human Albumin Enzyme-Linked Immunosorbent Assay (ELISA) kit (Catalog No. E88-129; Bethyl Laboratories, Montgomery, TX, United States), according to the manufacturer’s instructions. Absorbance was measured using a microplate reader (SILFA; BioTek Instruments Inc., Winooski, VT, United States).

### 2.5 RNA extraction and quantitative real-time polymerase chain reaction

Total RNA was isolated using TRIzol reagent (Catalog No. 1596018; Invitrogen, Waltham, MA, United States) and eluted in 20 μL diethyl pyrocarbonate–treated water (Catalog No. 46-2224; Invitrogen). Total RNA concentration and purity were measured using a Nanodrop™ One system (Catalog No. ND-ONE; Thermo Fisher Scientific, Waltham, MA, United States). Complementary DNA was synthesized from 1 μg of total RNA using TOPscript™ RT Dry Mix (dT18) (Catalog No. RT201; Enzynomics, Daejeon, Republic of Korea) and a Veriti™ Dx 96-well Thermal Cycler (Catalog No. 4452300; Applied Biosystems, Waltham, MA, United States). Quantitative real-time polymerase chain reaction was performed using SYBR™ Green Polymerase Chain Reaction Master Mix (Catalog No. 4309155; Applied Biosystems) with a 7,500 Fast Real-Time Polymerase Chain Reaction System (Applied Biosystems). The cycle threshold value for each target gene was determined according to the manufacturer’s instructions. Relative gene expression was quantified using the ΔΔCt method and was normalized to that of *β-actin*. The primer sequences used are listed in Supplementary Table S2.

### 2.6 Indocyanine green uptake and release

To evaluate indocyanine green (ICG) uptake and release, liver organoids were first washed with cold phosphate-buffered saline (PBS) to remove Matrigel residue, and then incubated with ICG (1 mg mL^-1^: Catalog No. I2633; Sigma–Aldrich, St. Louis, MO, United States) for 15 min at 37 °C in a 5% CO_2_ incubator. Images were acquired using a microscope (DM 3000; Leica Microsystems; Wetzlar, Germany). Thereafter, organoids were gently rinsed thrice with PBS and supplied with fresh medium. After 1 h of incubation under the same conditions, images of ICG release were collected.

### 2.7 Immunofluorescence assay

For immunofluorescence (IF) analysis, differentiated organoids were seeded onto a Nunc Lab-Tek II Chamber Slide System (Catalog No. 154461PK; Thermo Fisher Scientific). After differentiation, organoids were washed thrice with PBS and fixed in 10% neutral buffered formalin at 4 °C for 1 h. Fixed organoids were then washed thrice with PBS containing 0.1% Tween® 20 (PBS-T). Permeabilization was performed using 0.1% Triton X-100 for 15 min, followed by three washes with PBS-T. Cells were blocked with 4% bovine serum albumin at room temperature (15–25 °C) for 1 h.

The antibodies used in this study are listed in Table 5. The organoids were incubated overnight at 4 °C with the primary antibody. After three additional washes with PBS-T, secondary antibody incubation was conducted for 1 h at room temperature under dark conditions. Afterward, the slides were rinsed thrice with PBS-T, the chamber mold was removed, and the 4′,6-diamidino-2-phenylindole mounting solution (Catalog No. H-1200-10; Vector Laboratories Inc., Newark, CA, United States) was applied for 5 min. Fluorescent images were acquired using a Leica Thunder Imager (Leica Microsystems).

**TABLE 5:** Antibodies used in this study.

Antibodies	Cat no.	Company	Dilution
anti-albumin	A80-129A	Bethyl laboratories	1:1,000
anti-HNF4α	3113s	Cell signaling technology	1:1,500
Donkey anti-Goat IgG (H + L) Cross-Adsorbed secondary antibody, Alexa Fluor 488	A11055	Thermo Fisher	1:2,000
Goat anti-Rabbit IgG (H + L) Highly Cross-Adsorbed secondary antibody, Alexa Fluor plus 594	A32740	Thermo Fisher	1:2,000

### 2.8 Analysis of cell viability

Liver organoid differentiation was performed as previously described (Choi et al., 2024). Differentiation status was assessed using ALB secretion and CYP3A4 mRNA expression. The organoids were seeded onto U-bottom 96-well plates (Catalog No. 34296; SPL Life Sciences, Pocheon, Republic of Korea) and stabilized for 48 h. Subsequently, they were exposed to the chemicals once daily for 5 days. Chemical stocks were prepared in DMSO (Catalog No. sc-358801; Santa Cruz Biotechnology, Dallas, TX, United States) or DW and diluted in the culture medium to the desired concentrations (Supplementary Table S1). The concentration of the final vehicle control (DMSO or DW) did not exceed 1% under any conditions. On day 5 of treatment, chemical-treated supernatants were collected and stored at −80 °C for further analysis. Cell viability was assessed using an EZ-Cytox reagent assay (Catalog No. EZ-1000; DoGenBio, Seoul, Republic of Korea), which reflects cellular metabolic activity based on intracellular reducing capacity, according to the manufacturer’s protocol. Absorbance was measured at 450 nm using a microplate reader (SILFA; BioTek Instruments Inc.). Cell viability was calculated relative to the untreated control, as follows:

$$Cell\;viability\;\left(\%\right)=\frac{sample-blank}{control-blank}\times 100$$ (1)

The half-maximal toxic concentration (TC_50_) was determined by plotting the logarithm of the compound concentration against cell viability, and the data were fitted using the following equation:

$$Y=\frac{100}{\left[1+10^{\left(\left(\log{TC}_{50}-x\right)\times hill\;slope\right)}\right]}$$ (2)

### 2.9 Bright-field imaging of chemical-treated organoids

Chemical treatment was performed as described in Section 2.8. Briefly, human hepatic organoids were seeded at approximately 5–6 organoids per well in 96-well U-bottom plates and allowed to stabilize for 48 h. To prevent focus drift, the medium was refreshed once daily by gently aspirating from the well edge and replacing it with medium containing the same chemical concentration. Bright-field images were acquired on day 0, before treatment, and on day 5 post-treatment using a Leica Thunder Imager (Leica Microsystems).

### 2.10 α1-Antitrypsin ELISA

To measure α1-antitrypsin (AAT) secretion, culture supernatants were collected 24 h after medium replacement. Quantification was performed using a human AAT ELISA kit (Catalog No. EA5101-8; AssayPro, St. Charles, MO, United States) according to the manufacturer’s instructions. Absorbance was recorded using a microplate reader (S1LFA; BioTek Instruments, Inc.), and the values were normalized to the cell number.

### 2.11 Alanine aminotransferase and aspartate aminotransferase ELISA

To determine alanine aminotransferase (ALT) and aspartate aminotransferase (AST) levels, the culture medium was collected 24 h after medium replacement. Human ALT ELISA (Catalog No. ab234578; Abcam, Cambridge, United Kingdom) and Human AST ELISA kits (Catalog No. ab263881; Abcam) were employed following the manufacturer’s protocols. Absorbance was measured using a microplate reader (S1LFA; BioTek Instruments, Inc.).

### 2.12 RNA sequencing

RNA sequencing (RNA-seq) was performed by ROKIT Genomics (https://rokitgenomics.com; Seoul, Republic of Korea). Total RNA was extracted using TRIzol reagent (Invitrogen), and at least 1 μg of RNA per sample was used for library preparation. Each condition included three biological replicates (*n* = 3) generated from independently cultured organoids within the same differentiation batch. Sequencing was conducted to generate 150-bp paired-end reads with a minimum depth of 30 million reads per sample. Raw reads were quality-checked using FastQC (v0.12.11) and trimmed using Trimmomatic (v0.40). Clean reads were aligned to the human reference genome (Ensembl Release 114) using STAR (v2.7.11b), and gene and transcript abundances were quantified using RNA-Seq with Expectation-Maximization (v1.3.3). Gene-level counts and normalized expression values were imported using tximport (v1.30.0) and reported as fragments per kilobase of transcript per million mapped reads. Differential expression analysis was performed using DESeq2 (v1.42.1) based on raw counts with size-factor normalization. Genes with a false discovery rate (FDR) < 0.05 and |log2 fold change| ≥ 1 were considered significantly differentially expressed genes (DEGs). Functional enrichment analyses, including Gene Ontology (GO) over-representation and Kyoto Encyclopedia of Genes and Genomes (KEGG) pathway analyses, were conducted using clusterProfiler (v4.13.1), fgsea (v1.28.0), and msigdbr (v24.1.0). Functional network visualization was performed using ClueGO (v2.5.10) in Cytoscape (v3.9.1). K-means clustering (k = 5) and heatmap visualization were performed using pheatmap (v1.0.13). Raw sequencing data are available from the provider database at http://data.rokitgenomics.co.kr/sharing/6s6crFK2G.

### 2.13 Statistical analysis

All experiments were performed at least three times, as indicated in the figure legends. Statistical analyses were performed using GraphPad Prism (GraphPad Software, version 10). Data are presented as the mean ± standard deviations (SD). Comparisons of three or more groups were assessed with one-way anylsis of variance (ANOVA) followed by Tukey’s *post hoc* multiple comparison test. comparisons between two groups were analyzed with Student’s t-test. Statistical significance was defined as follow: *p < 0.05, **p < 0.01, ***p < 0.001, and ****p < 0.0001.

## 3 Results

### 3.1 Establishment of an organoid-based toxicity evaluation platform

**FIGURE 1:**
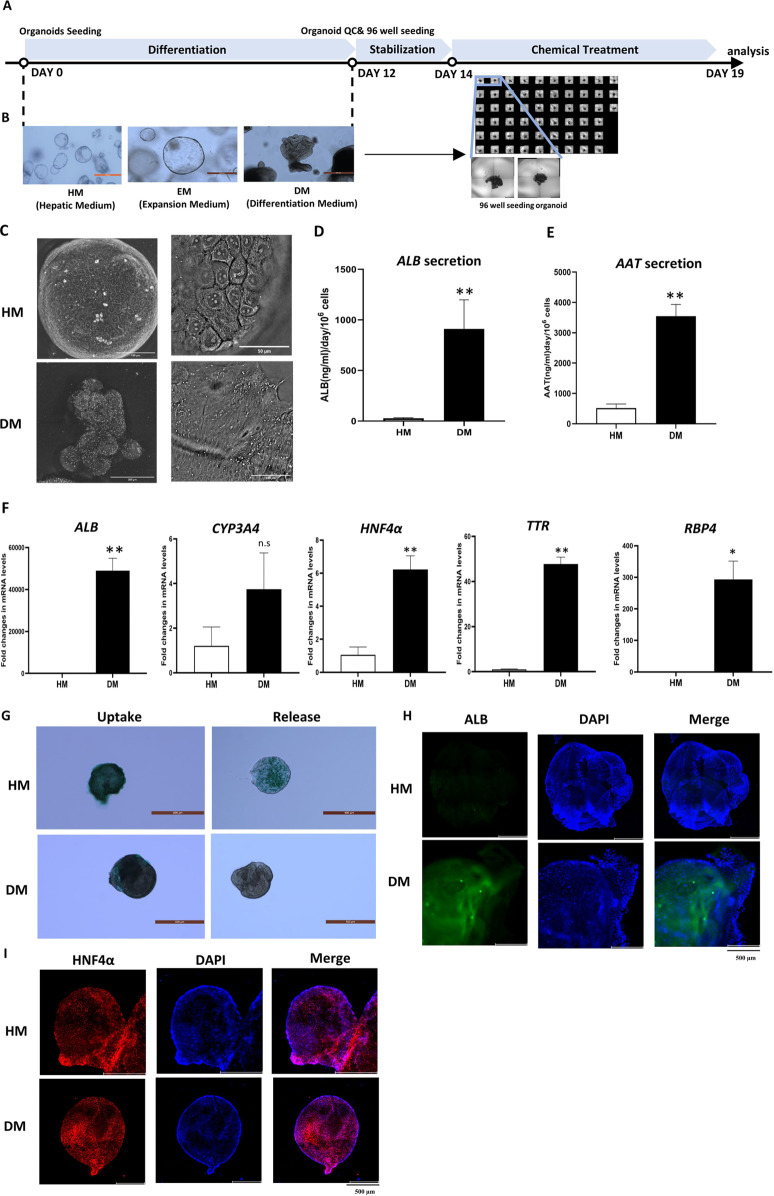
Functional validation of hepatic organoids and establishment of a platform for toxicity testing. **(A)** Schematic overview of the experimental workflow for hepatic organoid generation and chemical toxicity testing. **(B)** Representative bright-field images showing morphological transitions across culture stages in HM, EM, and DM. Scale bar = 500 µm. Images of differentiated organoids uniformly seeded into U-bottom 96-well plates for subsequent chemical treatment. Scale Bar = 1,000 µm. **(C)** Representative three-dimensional holotomography imaging of morphological differences before and after differentiation. Scale bar = 50 μm, 100 µm. Functional analyses comparing organoids before and after differentiation included **(D)** ALB expression, **(E)** AAT expression, and **(F)** mRNA expression of hepatocyte markers and drug-metabolizing genes. Data are presented as the mean ± standard error of the mean (*n* = 3) and were analyzed using Student’s *t*-test (*p < 0.05, **p < 0.01 versus HM). **(G)** Representative image of ICG uptake and release. Scale bar = 500 µm. **(H)** IF staining images of liver-specific markers for ALB (green) with nuclei counterstained using 4′,6-diamidino-2-phenylindole (blue). IF images were acquired at ×200 magnification with a scale bar of 500 µm. **(I)** IF staining images of HNF4α (red) and nuclei (blue). Scale bar = 500 µm. HM, hepatic medium; EM, expansion medium; DM, differentiation medium; ALB, albumin; AAT, α1-antitrypsin; IF, immunofluorescence; ICG, indocyanine green.

Hepatic organoids were generated from pluripotent stem cells as described previously (Mun et al., 2023; Mun et al., 2019). To promote hepatic specification, proliferation, and maturation, the organoids were sequentially cultured in defined media (Tables 1–3). The differentiation process involved an initial exposure to HM and EM for 3 days to promote hepatocyte proliferation, followed by cultivation in DM for an additional 8 days (Figure 1A). The representative morphological features of the organoids at each culture stage are shown in Figure 1B. HM organoids exhibited cystic structures with transparent lumens and thin epithelial layers. EM organoids displayed expanded spherical morphologies, and DM organoids developed compact folded structures with darker lumens, consistent with hepatocyte-like differentiation. On day 8 of differentiation, hepatic functionality was verified based on the evaluation criteria outlined in established organoid guidelines (Moon et al., 2024). Functionally mature organoids were uniformly seeded in U-bottomed 96-well plates and stabilized for 48 h. Next, the organoids were exposed to the test chemicals once daily for five consecutive days, with fresh media containing the chemicals replaced at each exposure (Figures 1A,B).

To confirm the establishment and successful implementation of hepatic organoid characteristics and functions, various functional assessments were conducted on day 8 of differentiation. First, we confirmed distinct morphological transitions associated with successful differentiation. 3D holotomography imaging was conducted to examine the morphological changes in hepatic organoids before and after differentiation. Organoids cultured in HM exhibited smooth, spherical, and cystic structures with transparent interiors and thin epithelial layers. Because of their low cellular density and transparent morphology, individual cells and nuclei were distinguishable, exhibiting uniform size and even distribution characteristic of an immature hepatic progenitor stage (Figure 1C). In contrast, organoids cultured in DM were smaller and more compact, with pronounced folding of the outer layer and increased cellular aggregation. The cell nuclei appeared less distinct and were surrounded by densely organized cytoplasmic structures, reflecting enhanced tissue organization and intercellular adhesion associated with hepatic maturation. The 3D holotomography imaging effectively demonstrated that organoids morphologically recapitulated the key features of hepatic differentiation (Figure 1C) (Sorrentino et al., 2020; StemCell Technologies, 2024; Ye et al., 2020).

Previous studies have identified ALB, HNF4α, and transthyretin (TTR) as representative markers associated with hepatocyte maturation (DeLaForest et al., 2011; Du et al., 2018). Accordingly, the expression of liver-specific markers, including ALB and AAT, increased in differentiated organoids compared with that under HM culture conditions (Figures 1D,E). Additionally, we assessed the mRNA expression levels of mature hepatocyte markers (*HNF4α*, *ALB*, and *TTR*) and the drug-metabolizing gene *CYP3A4*. As shown in Figure 1F, DM organoids exhibited significantly higher expression levels of these hepatic and metabolic genes than those in HM organoids, suggesting hepatic maturation.

Liver organoids cultured under both HM and DM conditions exhibited distinct ICG uptake, as evidenced by a dark green coloration, followed by progressive decolorization upon release, reflecting liver-specific uptake and excretory functions (Figure 1G). IF staining confirmed the expression of hepatic proteins in the differentiated organoids. IF analysis indicated significantly increased ALB expression in DM compared with that in the HM group within the organoid (Figure 1H). However, HNF4α expression remained consistently high in both HM and DM groups (Figure 1I).

### 3.2 Analysis of viability, albumin secretion, and morphological disruption in chemical-treated hepatic organoids

**FIGURE 2:**
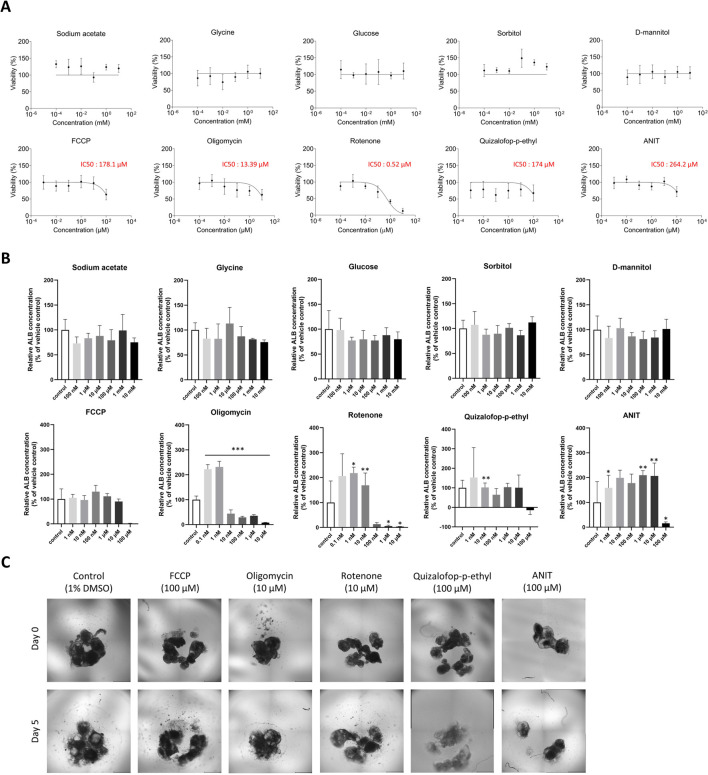
Assessment of cell viability, ALB secretion, and morphological changes following chemical treatment of liver organoids. Liver organoids were treated with 10 chemical compounds (5 toxic and 5 non-toxic) for 5 days. **(A)** Cell viability and **(B)** ALB secretion are presented as percentage change relative to the vehicle control. Treatment concentrations were selected based on solubility tests for each compound. Cell viability data are shown as mean ± standard deviation (*n* = 3). ALB secretion was analyzed using one-way analysis of variance followed by Tukey’s *post hoc* test, comparing each treatment group with the vehicle control group. *p < 0.05, **p < 0.01, and ***p < 0.001. **(C)** Morphological changes were observed before and after treatment. All images were captured at ×100 magnification with a scale bar of 500 μm. ALB, albumin.

Following confirmation of chemical solubility, liver organoids were treated with the prepared concentration range for 5 days. Subsequent analyses of cell viability and liver function markers were performed to assess concentration-dependent toxicity responses. Accordingly, cell viability was calculated using Equation 1, and TC50 values were determined using Equation 2, indicating distinct cytotoxicity differences between the two groups (Figure 2A). The five toxic chemicals exhibited lower TC50 values (FCCP = 178.1 µM, oligomycin = 13.3 µM, rotenone = 0.517 µM, quizalofop-p- ethyl = 174 µM, and ANIT = 264.2 µM) than those of the non- hepatotoxic substances (for which TC50 values were not detectable within the tested concentration range).

ALB, a liver-specific functional biomarker, is a major plasma protein synthesized exclusively by hepatocytes (O'Brien et al., 2000). A reduction in ALB secretion reflects functional impairment in hepatocytes. Therefore, we analyzed the culture medium at the same time point as the cell viability measurements to assess ALB secretion levels. Toxic chemicals induced a significant decrease in ALB secretion, whereas non-toxic chemicals caused no significant changes. In both cases, trends in ALB secretion closely mirrored those observed for cell viability (Figure 2B).

To investigate the correlation between cell viability and ALB secretion, we performed Pearson’s correlation analysis and calculated *p*-values. The results indicated a strong correlation for three of the five toxic chemicals, namely FCCP (*r* = 0.845), oligomycin (0.894), and rotenone (0.785), indicating a strong positive relationship between cell viability and ALB secretion (Supplementary Figure S3). Comparison of the reduction in cell viability and ALB secretion in the highest-concentration treatment groups of the five compounds relative to those of the control revealed a decrease in ALB secretion alongside changes in cell viability for all chemicals, except quizalofop-p-ethyl (Supplementary Figure S3A).

Bright-field microscopy showed consistent morphological features across the control and toxicant-treated organoids on day 0 (pretreatment). Organoids exhibited compact, opaque aggregates with irregular yet well-defined boundaries and dense cellular clustering regardless of subsequent treatment allocation (Figure 2C). By day 5, distinct morphological differences emerged in the specific toxicant-treated organoids compared with the control (Figure 2C). FCCP treatment (100 µM) resulted in extensive cellular debris, appearing as scattered dark puncta surrounding the aggregates, a feature absent in the control. The organoid boundary, which was initially well-defined on day 0, became irregular, jagged, and partially effaced after treatment, reflecting the loss of structural integrity. Rotenone treatment (10 µM) induced a prominent swelling phenotype, with organoids appearing enlarged and distended compared with the compact control aggregates. This is consistent with intracellular edema or impaired volume regulation. In contrast, organoids treated with oligomycin, quizalofop-p-ethyl, or ANIT exhibited no discernible morphological alterations relative to the control at this resolution, despite possible underlying functional changes.

### 3.3 Hepatic functional assessment of toxic chemicals on liver organoids

Based on the initial screening results, FCCP, oligomycin, and rotenone were selected for further functional assessment, including AAT secretion, hepatic gene expression, ICG uptake and release, and IF analysis. Pearson’s correlation analysis between cell viability and ALB secretion revealed a compound-dependent relationship between the two endpoints.

**FIGURE 3:**
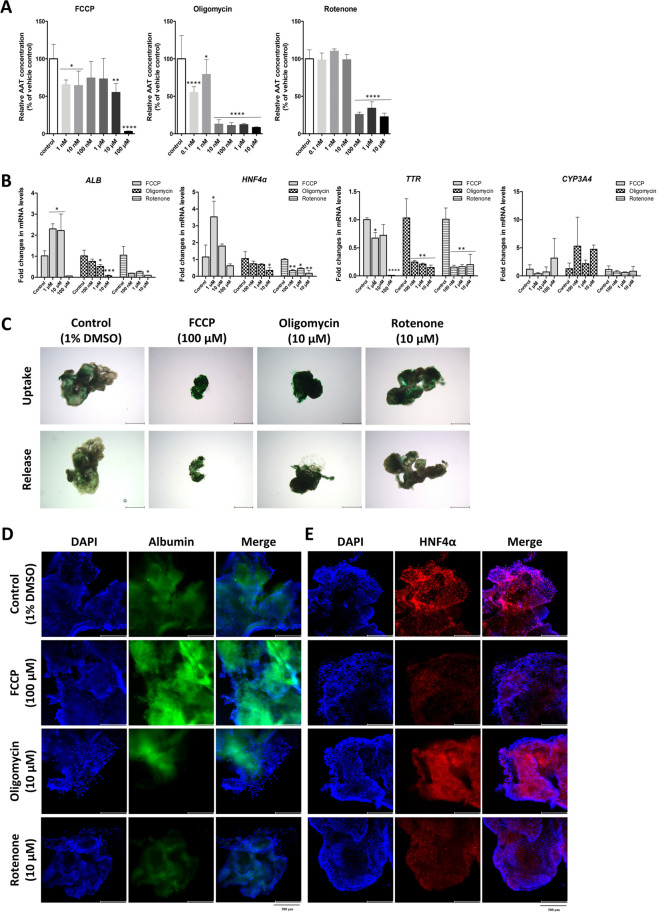
Assessment of AAT secretion, hepatic gene expression, ICG uptake and release, and functional hepatic markers in liver organoids following chemical treatment. **(A)** AAT secretion and **(B)** mRNA expression levels of liver function markers (*ALB*, *HNF4α*, *TTR,* and *CYP3A4*) in hepatic organoids following treatment with FCCP, oligomycin, and rotenone. Data are presented as the mean ± standard deviation (*n* = 3) and were analyzed using one-way analysis of variance followed by Tukey’s *post hoc* test, with each treatment group compared with the vehicle control. *p < 0.05, **p < 0.01, ***p < 0.001, and ****p < 0.0001. **(C)** Representative images of ICG uptake and release following treatment with the three chemicals. Scale bar = 500 µm. IF staining imaging was performed for **(D)** ALB (green), **(E)** HNF4α (red), and nuclei (4′,6-diamidino-2-phenylindole). IF images were captured at ×200 magnification; scale bar = 500 µm. AAT, α1-antitrypsin; ICG, indocyanine green; FCCP, carbonyl cyanide 4-(trifluoromethoxy)phenylhydrazone; IF, immunofluorescence; ALB, albumin.

AAT is a key serine protease inhibitor that regulates neutrophil elastase, which is synthesized in hepatocytes (Xiang et al., 2025). AAT deficiency causes pulmonary and hepatic injury, as well as immune regulation disruption (Blanco, 2017; Xiang et al., 2025). Therefore, AAT was used as an indicator of liver damage. AAT levels were significantly decreased following FCCP, oligomycin, and rotenone treatment at concentrations above 10 μM, 0.1 nM, and 100 nM, respectively, compared with the control group (Figure 3A).

To assess hepatocyte impairment and the functional effects of the three chemicals on liver organoids, we examined the mRNA expression levels of *ALB*, *HNF4α*, *TTR*, and *CYP3A4* (Figure 3B). As shown in Figure 3B, all three chemicals induced a dose-dependent decrease in *ALB* expression. Oligomycin and rotenone significantly reduced *ALB* expression relative to that in the control group. These findings are consistent with the ALB secretion results shown in Figure 2B. *HNF4α* and *TTR* expression exhibited a similar tendency to that of *ALB*. At the highest concentrations of the three chemicals, *TTR* expression levels were significantly reduced compared with those in the control. Treatment with FCCP, oligomycin, and rotenone reduced the mRNA expression of hepatocyte-specific genes, indicating decreased mRNA abundance at the sample level. In contrast, no significant increase in *CYP3A4* expression was observed among the three compounds (Figure 3B). Each compound generated a distinct pathway-level signature despite a shared mitochondrial inhibitory function.

ICG is an indicator of the interplay between sinusoidal uptake and biliary excretion (Bi et al., 2024). The functionality of liver organoids was further assessed using ICG uptake and release, as well as IF staining. Liver organoids treated with the three compounds efficiently absorbed and subsequently released ICG, and the resulting transport activity was not affected (Figure 3C). IF staining for ALB and HNF4α was performed after 5 days of treatment with the three chemicals. IF analysis showed a significant reduction in ALB in the oligomycin and rotenone groups compared with that in the control group (Figure 3D), consistent with the observed *ALB* mRNA expression levels (Figure 3B). However, FCCP showed higher ALB IF than that in the control group, which was inconsistent with the mRNA expression results. IF analysis of HNF4α revealed a lower intensity for all three chemicals compared with that for the control group (Figure 3E), consistent with *HNF4α* mRNA expression (Figure 3B). In the control group, HNF4α was predominantly localized in the nucleus. In contrast, in the chemically treated group, it appeared to be degraded and redistributed to the cytoplasm. The organoid model demonstrated the ability to show chemical-specific molecular signatures. However, the current results are insufficient to establish definitive mechanistic links and require further investigation.

### 3.4 Hepatic functional indicators and inflammation-related transcriptional responses

**FIGURE 4:**
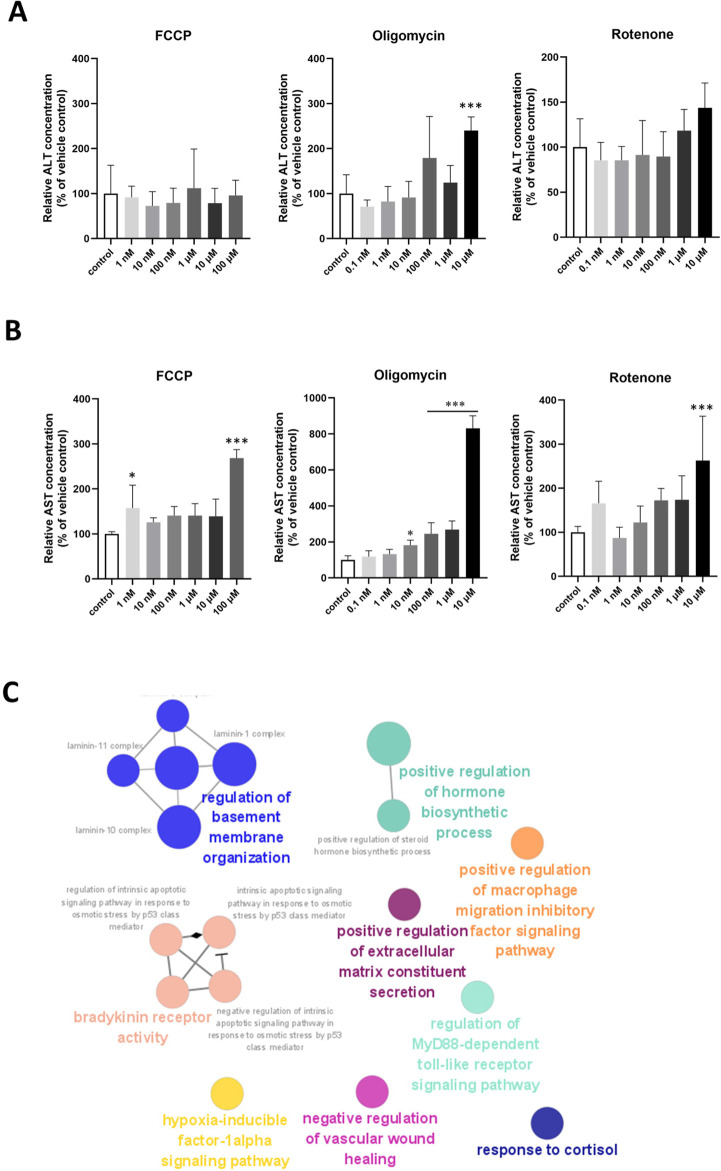
Functional injury markers and inflammation-related gene network in hepatic organoids. **(A)** ALT and **(B)** AST levels in hepatic organoids following treatment with FCCP, oligomycin, and rotenone. ALT and AST levels were analyzed using one-way analysis of variance followed by Tukey’s *post hoc* test, with each treatment group compared with the vehicle control. *p < 0.05, **p < 0.01, and ***p < 0.001. **(C)** Network visualization of inflammation-associated genes extracted from a k-means cluster enriched for immune- and stress-related genes. ALT, alanine aminotransferase; AST, aspartate aminotransferase; FCCP, carbonyl cyanide 4-(trifluoromethoxy)phenylhydrazone.

Liver injury is typically associated with increased release of ALT and AST, which are key indicators of hepatocellular damage (Lala, Zubair and Minter, 2025). Consistent with this expectation, all three tested chemicals induced increases in ALT or AST levels compared with those of the control group (Figures 4A,B). To further characterize the transcriptional responses to chemical exposure, k-means clustering (*k* = 5) was performed using DEGs at the highest treatment concentration for each chemical. Five distinct gene expression modules were identified (Supplementary Figure S4). One cluster exhibited coordinated alterations across a wide spectrum of biological processes commonly associated with hepatic injury. These included changes in pathways related to inflammatory signaling, immune-associated transcriptional pathways, cellular stress responses, hormonal modulation, extracellular matrix organization, and tissue remodeling.

The distribution of these pathway-level changes suggests that chemical treatment engages multiple layers of the hepatic response, rather than activating a single isolated mechanism. A complete list of genes belonging to this cluster is provided in Supplementary Table S3. Using genes from this cluster, an inflammation-related gene network was constructed, revealing interconnected nodes representing coordinated immune- and stress-associated transcriptional activities induced by chemical treatment (Figure 4C). Each compound generated a distinct pathway-level signature, even under identical exposure conditions. These results indicated that human hepatic organoids reflect functional hepatocellular injury through the increased release of ALT and AST and capture diverse transcriptional programs related to inflammation, stress adaptation, and tissue structural remodeling.

### 3.5 Transcriptomic profiling and functional evaluation of chemical toxicity in human hepatic organoids

**FIGURE 5:**
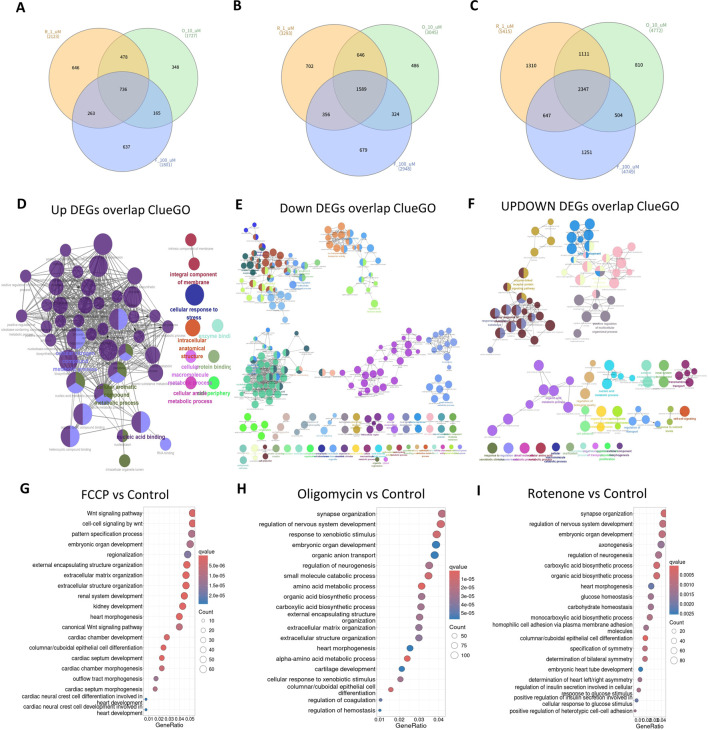
DEGs and functional enrichment profiles of FCCP-, oligomycin-, and rotenone-treated human hepatic organoids. Venn diagrams show the distribution of DEGs among the three compounds at the highest treatment concentrations. **(A)** Upregulated DEGs, **(B)** downregulated DEGs, and **(C)** all DEGs (up- and downregulated). R, rotenone; F, FCCP; O, oligomycin. **(D–F)** Network visualization of enriched processes derived from the DEG sets shown in panels **(A–C)**. **(G–I)** GO enrichment analysis for each compound. **(G)** FCCP-, **(H)** oligomycin-, and **(I)** rotenone-treated groups. DEGs, differentially expressed genes; FCCP, carbonyl cyanide 4-(trifluoromethoxy)phenylhydrazone.

To further evaluate transcriptional responses to compound exposure, we analyzed the molecular profiles of the organoids. We compared up- and downregulated DEGs at the highest treatment concentrations for each chemical. RNA-seq analysis showed compound-specific and shared transcriptional responses. The FCCP, oligomycin, and rotenone treatments resulted in 637, 348, and 646 uniquely upregulated genes, respectively. Meanwhile, 736 genes were induced across all treatments (Figure 5A). Similarly, 679, 486, and 702 genes were uniquely downregulated in FCCP-, oligomycin-, and rotenone-treated organoids, respectively, with 1,589 genes commonly suppressed across all conditions (Figure 5B). When considering both up- and downregulated genes, each treatment displayed a distinct DEG profile, with 1,251, 810, and 1,310 compound-specific DEGs, respectively, and 2,347 shared DEGs (Figure 5C). Concentration–response analysis showed a progressive increase in DEG counts across exposure levels for each treatment, supporting a dose-dependent expansion of the transcriptional response (Supplementary Figure S5).

Based on the DEG sets identified in the Venn diagram (Figures 5A–C), a network analysis was performed to examine the functional associations among the genes (Figures 5D–F). The upregulated DEG network (Figure 5D) showed densely connected clusters associated with cellular responses to stress, protein binding, and metabolic processes. Downregulated DEGs formed a large network consisting of modules related to metabolic regulation and catalytic activity. This included biosynthetic processes and nucleic acid binding, with additional peripheral clusters linked to cellular secretion and vesicle-mediated transport (Figure 5E). This pattern suggests the suppression of metabolic programs under chemical exposure.

The combined network, integrating both up- and downregulated DEGs (Figure 5F), formed a broad regulatory landscape composed of multiple modules, including cellular responses to stimuli, protein binding, catalytic activity, metabolic processes, and biosynthetic regulation. This indicated that both activated and suppressed pathways contribute to the transcriptional response. A list of genes belonging to this cluster is provided in Supplementary Tables S4–S6.

We conducted RNA GO analysis of organoids treated with hepatotoxic chemicals. FCCP treatment led to enrichment of pathways related to Wnt-mediated signaling and pattern specification, suggesting strong effects on developmental signaling programs (Figure 5G). Oligomycin treatment showed enrichment in pathways linked to xenobiotic response, transport activity, and amino acid and organic acid metabolism, reflecting adaptive metabolic responses (Figure 5H). In contrast, rotenone-enriched pathways were associated with neuronal organization and metabolic homeostasis, including synapse-related processes and carbohydrate regulation (Figure 5I). Overall, these results showed both shared and compound-specific transcriptional responses in a dose-dependent manner.

## 4 Discussion

In recent years, the application of organoid models has gained increasing attention. Human 3D organoid systems, which recapitulate the key structural and functional features of primary tissues through controlled maintenance and differentiation, are emerging as valuable platforms for studying human physiology and developmental processes. Compared with conventional 2D hepatocyte cultures, liver organoids better recapitulate the cellular heterogeneity, polarity, and microenvironment of the liver, enabling more physiologically relevant toxicity assessments (Afonso et al., 2024; Broutier et al., 2017; Verstegen et al., 2025). Although liver organoids have been used only sparingly in toxicity assessments of non-pharmaceutical chemicals, their application across diverse chemical classes has steadily increased (Alnasser, 2025; Bridgeman et al., 2025).

The applicability of *in vitro* models is often evaluated through comparisons with primary human hepatocytes, established hepatocyte cell lines, and *in vivo* toxicity data. In our previous study, the hepatic organoid model was evaluated against conventional *in vitro* systems and reference drug toxicity data, suggesting its applicability for exploratory toxicity assessment (Choi et al., 2024). That earlier work focused on pharmaceutical compounds and drug-induced toxicity responses. In contrast, the present study is the first to apply this organoid platform to chemical substances and to examine responses to selected chemicals classified under the Globally Harmonized System. Rather than establishing a fully validated predictive model, our focus was to determine whether the organoid system could detect toxicity-related responses across multiple endpoints under chemical exposure conditions. Upon exposure to 10 representative chemicals, the organoids exhibited concentration-dependent changes across functional, viability-related, and molecular readouts, enabling discrimination between toxic and non-toxic compounds.

In the present study, TC_50_ values were used as quantitative indicators of toxicity across selected reference compounds. In studies assessing dose–response relationships, TC_50_ values are commonly used as indicators of compound-induced cytotoxicity, with lower values reflecting higher toxicity. Recent studies have applied TC_50_ curves and values to evaluate responses in organoid-based systems. Our results showed that TC_50_ curves and values (dose–response patterns) varied depending on the chemical properties and toxicity profiles of the tested compounds. These observations are broadly consistent with patterns reported in other *in vitro* systems, although no direct comparison was performed in the current study. Furthermore, these findings suggest that the liver organoid model may differentiate compound-associated response patterns under the tested conditions (Ferguson et al., 2022; Figarola et al., 2015; Li et al., 2004; Seabra and Timbrell, 1994; Siddiqui et al., 2013). However, TC_50_ values, which were used as a primary measure of cytotoxicity, may not fully reflect the dose–response relationships relevant to human exposure. Incorporating benchmark dose modeling and comparisons with human exposure data would further enhance the translational relevance of the findings.

Pearson’s correlation analysis was performed to evaluate the relationship between cell viability and ALB secretion. The results indicated a strong correlation (*r* ≥ 0.75) for FCCP, oligomycin, and rotenone. ANIT and quizalofop-p-ethyl showed relatively weak correlations between cell viability and ALB secretion compared with the other toxicants. This weaker correlation reflects differences in hepatotoxic mechanisms rather than experimental viability. Although their primary hepatotoxic mechanisms differ, both compounds elicit cholestatic or cholestasis-like injury, which is characterized by early disturbances in bile transport, mitochondrial function, and metabolic homeostasis. These early functional impairments can reduce protein synthesis and secretion before detectable cell death, resulting in the dissociation of functional viability (Biserni et al., 2019; Elefsiniotis et al., 2007; Ferguson et al., 2022; Padda et al., 2011; Tanaka et al., 2009).

Given previous reports demonstrating that hepatic organoids can recapitulate cholangiocyte-like cells, cholangiocyte-specific endpoints (e.g., GGT) merit further discussion (Kim et al., 2023). However, as EZ-Cytox primarily reflects cellular metabolic activity rather than direct viability, the observed decreases should be interpreted with caution, as they may partly reflect alterations in cellular metabolism, particularly under mitochondrial toxic conditions (Ghasemi et al., 2021). In addition, morphological assessment showed that hepatic organoids exposed to hepatotoxic compounds exhibited minimal structural alterations. Although certain treatments induced slight irregularities or compaction, most toxic compounds did not result in any noticeable morphological changes. The overall spherical and organized architecture was largely maintained. These findings suggest that morphological changes in hepatic organoids are relatively limited, even under toxic conditions. Therefore, morphological assessment alone may not fully capture compound-induced effects, highlighting the value of evaluating multiple endpoints, including cell viability and ALB secretion.

FCCP, oligomycin, and rotenone were selected for further functional characterization. Following treatment with these compounds, functional impairment of liver organoids was evident at both the molecular and cellular levels. Similar to ALB secretion, toxic chemical–treated organoids showed decreased AAT secretion in a dose-dependent manner. However, the mRNA expression of hepatocyte-specific genes was not consistently reduced; *ALB*, *HNF4α*, and *TTR* exhibited reduced transcript abundance, whereas *CYP3A4* expression showed variable expression. The heterogeneous patterns observed in *CYP3A4* expression may reflect the complex relationship between transcriptional regulation and enzymatic activity (Hakkola et al., 2020). In particular, mRNA expression levels do not necessarily correlate with CYP3A4 enzyme function, as substrate specificity and transcriptional induction are governed by distinct regulatory mechanisms. For example, while rotenone is a known substrate of CYP3A4, FCCP and oligomycin are not reported to be CYP3A4 substrates (Marta Ferrati, 2023). Therefore, the absence of significant changes in *CYP3A4* mRNA levels does not preclude metabolic activity, and further functional assays are required to assess CYP enzyme activity.

AAT is synthesized in the endoplasmic reticulum of hepatocytes, processed through the Golgi apparatus, and secreted into the extracellular space via an ATP-dependent pathway (Karatas and Bouchecareilh, 2020). Therefore, efficient AAT secretion requires intact mitochondrial energy metabolism. All three selected compounds—FCCP, oligomycin, and rotenone—are well-established mitochondrial toxicants that inhibit ATP synthesis through distinct mechanisms, including dissipation of the mitochondrial membrane potential or inhibition of the electron transport chain (Chiu et al., 2019; Li et al., 2003; Ratajczak et al., 2019). Therefore, the observed reduction in AAT secretion in the present study is consistent with mitochondrial dysfunction–induced energy depletion. This likely impairs ATP-dependent protein synthesis, intracellular trafficking, and secretion processes (Martínez et al., 2020; Stephens, 1998). This interpretation is further supported by previous reports demonstrating that FCCP induces intracellular protein accumulation and aggregation by collapsing mitochondrial membrane potential, suppressing ATP production, and disrupting ATP-dependent protein transport and secretion pathways (Guillaud et al., 2025; Vekaria et al., 2025).

Consistent with these findings, IF analysis revealed compound-specific responses, with distinct staining patterns observed depending on the tested compounds. FCCP treatment resulted in intracellular accumulation of ALB despite decreased secretion levels, whereas oligomycin induced only a mild reduction in ALB intensity without evident retention. Rotenone treatment, in contrast, led to decreased ALB levels. Notably, ICG uptake and release were not significantly affected. The decrease in AAT secretion and intracellular retention of hepatocyte proteins detected by IF analysis suggests that mitochondrial ATP depletion impairs ATP-dependent protein transport and secretion pathways in chemically treated organoids (Guillaud et al., 2025; Vekaria et al., 2025). In contrast, physiological processes such as ICG handling were less sensitive to damage, highlighting the differential vulnerability of hepatic functions within the organoid system.

Furthermore, exposure to the three compounds increased ALT and AST in the media, indicating acute hepatocellular injury in human hepatic organoids. As elevated transaminase levels represent a primary clinical indicator of liver damage, these findings suggest that the organoid model may reflect aspects of hepatocellular injury responses under the tested conditions (Lala et al., 2025). In addition to functional changes, transcriptional analysis was performed at concentrations at which ALB secretion was markedly reduced, enabling comparative evaluation of functional impairment across compounds. Transcriptomic clustering revealed coordinated activation of pathways related to inflammation, immune-related signaling, and cellular stress responses. These inflammation-associated gene networks likely reflect stress-responsive transcriptional programs in hepatocytes; however, further studies are required to clarify their biological significance.

Previous studies have shown that hepatotoxic compounds can induce transcriptional responses related to cellular stress, inflammation, and metabolic adaptation in liver-derived systems (Ter Braak et al., 2021; Wijaya et al., 2024), consistent with the pathway-level changes observed in the present study. However, for mitochondrial Toxicants, such as FCCP and oligomycin, prior studies have primarily focused on bioenergetic dysfunction and cytotoxicity rather than comprehensive transcriptomic profiling, limiting direct comparison. These findings suggest that organoid-based RNA profiling may be useful for exploring compound-associated transcriptional responses under the tested conditions. In the present study, transcriptomic analysis was used to characterize RNA-level changes associated with compound exposure rather than to derive detailed mechanistic insights. Therefore, the observed transcriptomic responses should be interpreted cautiously, as further studies are required to determine their mechanistic relevance. Overall, the results suggest that hepatic organoids exhibit functional and transcriptional responses to chemical exposure and may serve as an exploratory *in vitro* platform for investigating compound-associated effects.

Venn analysis showed that FCCP, oligomycin, and rotenone induced distinct sets of up- and downregulated genes while sharing a substantial common response. The concentration-dependent increase in the number of DEGs supports a graded transcriptional response to exposure. Network analyses indicated that upregulated genes were enriched in stress-adaptive functions. Meanwhile, downregulated genes were associated with metabolic programs. GO analysis highlighted distinct mechanistic differences: FCCP activated Wnt-related developmental signaling and oligomycin-induced xenobiotic and metabolic compensation pathways, while rotenone affected neuronal organization and metabolic homeostasis. These compound-specific signatures indicate differences in transcriptional responses among the tested compounds. This supports the use of organoid-based RNA profiling for mechanistic classification and comparative toxicity assessment. Furthermore, the findings suggest that organoid-based RNA profiling can be applied to explore compound-specific transcriptional responses.

Notably, the deeper analyses in our study were conducted on a subset of compounds with related mechanisms of action, primarily mitochondrial toxicants (FCCP, oligomycin, and rotenone). Therefore, the current findings are inherently biased toward mitochondrial dysfunction and do not capture the broader mechanistic diversity of hepatotoxicants. Overall, this study underscores the potential of human hepatic organoids as a promising and scalable platform for an exploratory *in vitro* model. Continued optimization and integration of multiparametric readouts are essential to realize the full potential of human hepatic organoids as next-generation models for human-relevant chemical safety assessments.

The selected compounds were intended as a pragmatic exploratory set of reference chemicals with known toxicity profiles for an exploratory feasibility evaluation of the organoid platform across multiple endpoints. Nonetheless, this study has several limitations, including the restricted number of tested chemicals, the lack of direct mechanistic validation, and the incomplete representation of liver cellular composition and chronic exposure conditions. In addition, although the organoids contain hepatocyte-, cholangiocyte-, and hepatic stellate-like populations, further characterization of additional supporting cell types remains necessary. Furthermore, the selected compounds were not intended to represent the full diversity of hepatotoxic mechanisms, and broader compound classes should be included in future studies.

## 5 Conclusion

Toxicity responses to reference compounds were evaluated using multiple endpoints, including viability, functional markers, and transcriptomic analysis, to assess compound-associated effects in hepatic organoids. The tested compounds showed compound-specific response patterns, including dose-dependent reductions in cell viability and ALB secretion. Transcriptomic analysis further revealed distinct gene expression changes associated with hepatotoxic responses across different treatment conditions. The present study was designed as an exploratory feasibility evaluation using selected reference compounds under the tested conditions. Although these findings suggest the potential utility of hepatic organoids as an *in vitro* platform for an exploratory *in vitro* model, the limited chemical diversity, lack of functional metabolic validation (e.g., CYP activity), and absence of direct comparison with *in vivo* data or established models indicate the need for further studies to assess broader applicability, predictive performance, and exposure relevance. Collectively, this exploratory study suggests that hepatic organoids may serve as an exploratory *in vitro* model for assessing compound-associated responses, although further validation is required.

## Data Availability

The datasets presented in this study can be found in online repositories. The names of the repository/repositories and accession number(s) can be found in the article/Supplementary Material.
