# Supplementary material for: A human liver organoid platform for hepatotoxicity assessment: evaluation using reference compounds
Source: Front Toxicol. 2026 Jun 15;8:1805474. doi: 10.3389/ftox.2026.1805474 (PMC13310563; doi:10.3389/ftox.2026.1805474)
Supplement: Supplementary file 1 [file Table1.docx]

**Table S1. Solubility results from ten chemical compounds**

| **Solvent** | **Stock solution conc.** | **1 M** | **0.1 M** | **10 mM** | **1 mM** | **Final Conc.** | |
| --- | --- | --- | --- | --- | --- | --- | --- |
|  | **Working solution conc.** | **10 mM** | **1 mM** | **0.1 μM** | **10 μM** | **Stock Conc.** | **Working Conc.** |
| DMSO | FCCP | X | X | O |  | 1 mM | 10 μM |
|  | Quizalofop-p-ethyl | X | X | O |  | 10 mM | 100 μM |
|  | Rotenone | X | X | X | O | 1 mM | 10 μM |
|  | ANIT | X | X | O |  | 10 mM | 100 μM |
|  | Oligomycin | X | X | X | O | 1 mM | 10 μM |
| DW | Sodium acetate | O |  |  |  | 1 M | 10 mM |
|  | D-mannitol | O |  |  |  | 1 M | 10 mM |
|  | Glucose | O |  |  |  | 1 M | 10 mM |
|  | Sorbitol | O |  |  |  | 1 M | 10 mM |
|  | Glycine | O |  |  |  | 1 M | 10 mM |

○ : Soluble X : Insoluble

**Table S2. List of PCR oligo primer sequences.**

| Name | Sequence (5’ → 3’, Forward) | Sequence (3’ → 5’, Reverse) |
| --- | --- | --- |
| ALB | TTTATGCCCCGGAACTCCTTT | AGTCTCTGTTTGGCAGACGAA |
| CYP3A4 | CTTCATCCAATGGACTGCATAAAT | TCCCAAGTATAACACTCTACACAGACAA |
| HNF4α | GGCCAAGTACATCCCAGCTTT | CAGCACCAGCTCGTCAAGG |
| TTR | TGGGAGCCATTTGCCTCTG | AGCCGTGGTGGAATAGGAGTA |
| BSEP | AGGGAGCTACCAGGATAGTTTAAGG | TCGTGCACCAGGTAAGAAAGC |
| β-actin | CATGTACGTTGCTATCCAGGC | CTCCTTAATGTCACGCACGAT |

**Table S3. List of differentially expressed genes (DEGs) in cluster 2**

| ID | Term | Associated Genes Found |
| --- | --- | --- |
| GO:0000915 | actomyosin contractile ring assembly | [ANLN, ECT2, RACGAP1] |
| GO:0001641 | group II metabotropic glutamate receptor activity | [GRM2, GRM3] |
| GO:0034124 | regulation of MyD88-dependent toll-like receptor signaling pathway | [CD300A, IRF1, IRF7] |
| GO:0051414 | response to cortisol | [IGFBP7, SLIT2, SLIT3] |
| GO:0061044 | negative regulation of vascular wound healing | [SERPINE1, SLC12A2, TNF] |
| GO:0061046 | regulation of branching involved in lung morphogenesis | [BMP4, SOX9, TNF] |
| GO:0061304 | retinal blood vessel morphogenesis | [COL4A1, FZD4, LAMA1] |
| GO:0070553 | nicotinic acid receptor activity | [HCAR2, HCAR3] |
| GO:0070557 | PCNA-p21 complex | [CDKN1A, PCNA] |
| GO:0007168 | receptor guanylyl cyclase signaling pathway | [GUCA1B, GUCY2D, NPPB, NPR1, NPR2, NPRL2] |
| GO:0071477 | cellular hypotonic salinity response | [SLC12A6, TRPV4] |
| GO:0072139 | glomerular parietal epithelial cell differentiation | [CD24, FOXJ1, PROM1] |
| GO:0003150 | muscular septum morphogenesis | [BMP4, FZD2, TGFBR3] |
| GO:0090270 | regulation of fibroblast growth factor production | [HEG1, PTGS2, RGCC] |
| GO:0090362 | positive regulation of platelet-derived growth factor production | [PTGS2, SERPINB7] |
| GO:0097411 | hypoxia-inducible factor-1 alpha signaling pathway | [HIF1A, PDK1, PDK3] |
| GO:0140361 | cyclic-GMP-AMP transmembrane import across plasma membrane | [LRRC8C, LRRC8E, SLC19A1] |
| GO:0008419 | RNA lariat debranching enzyme activity | [CWF19L1, DBR1] |
| GO:1900141 | regulation of oligodendrocyte apoptotic process | [AKAP12, GAS6] |
| GO:0003331 | positive regulation of extracellular matrix constituent secretion | [BMP2, IER3IP1, RGCC] |
| GO:2000097 | regulation of smooth muscle cell-matrix adhesion | [EFEMP2, PLAU, SERPINE1] |
| GO:2000405 | negative regulation of T cell migration | [CD200, CD200R1, RIPOR2] |
| GO:2000448 | positive regulation of macrophage migration inhibitory factor signaling pathway | [CD74, CXCR4] |
| GO:0008510 | sodium:bicarbonate symporter activity | [SLC4A5, SLC4A7, SLC4A8] |
| GO:0008526 | phosphatidylinositol transfer activity | [C2CD2L, OSBPL2, PITPNC1, PITPNM3, PLTP, TNFAIP8L3] |
| GO:0014034 | neural crest cell fate commitment | [EDN1, SFRP1, SOX9] |
| GO:0015677 | copper ion import | [SLC31A1, STEAP3, STEAP4] |
| GO:0046946 | hydroxylysine metabolic process | [PLOD2, PLOD3] |
| GO:0046947 | hydroxylysine biosynthetic process | [PLOD2, PLOD3] |
| GO:0006561 | proline biosynthetic process | [OAT, PYCR1, PYCR2] |
| GO:0055129 | L-proline biosynthetic process | [OAT, PYCR1, PYCR2] |
| GO:0071440 | regulation of histone H3-K14 acetylation | [GATA3, PIWIL2, WBP2] |
| GO:0071442 | positive regulation of histone H3-K14 acetylation | [GATA3, PIWIL2, WBP2] |
| GO:0046886 | positive regulation of hormone biosynthetic process | [ADM, BMP6, HIF1A, MIA3, VDAC2, WNT4] |
| GO:0090031 | positive regulation of steroid hormone biosynthetic process | [BMP6, VDAC2, WNT4] |
| GO:1904863 | regulation of beta-catenin-TCF complex assembly | [DACT1, SOX9] |
| GO:1904864 | negative regulation of beta-catenin-TCF complex assembly | [DACT1, SOX9] |
| GO:0021966 | corticospinal neuron axon guidance | [SCN1B, SLIT2] |
| GO:0021957 | corticospinal tract morphogenesis | [EPHA4, FBXO45, SCN1B, SLIT2] |
| GO:0032594 | protein transport within lipid bilayer | [ATP1B1, CD24, REEP2, RILPL2] |
| GO:0032596 | protein transport into membrane raft | [ATP1B1, CD24, REEP2] |
| GO:0004947 | bradykinin receptor activity | [BDKRB1, BDKRB2] |
| GO:1990127 | intrinsic apoptotic signaling pathway in response to osmotic stress by p53 class mediator | [BDKRB1, BDKRB2] |
| GO:1902238 | regulation of intrinsic apoptotic signaling pathway in response to osmotic stress by p53 class mediator | [BDKRB1, BDKRB2] |
| GO:1902239 | negative regulation of intrinsic apoptotic signaling pathway in response to osmotic stress by p53 class mediator | [BDKRB1, BDKRB2] |
| GO:0036305 | ameloblast differentiation | [BMP2, BMP4, ENAM, FST] |
| GO:0061209 | cell proliferation involved in mesonephros development | [BMP2, BMP4, GATA3, GPC3] |
| GO:0003134 | endodermal-mesodermal cell signaling involved in heart induction | [BMP2, BMP4] |
| GO:0003130 | BMP signaling pathway involved in heart induction | [BMP2, BMP4] |
| GO:0072138 | mesenchymal cell proliferation involved in ureteric bud development | [BMP2, BMP4, GPC3] |
| GO:0005606 | laminin-1 complex | [LAMA1, LAMB1, LAMC1] |
| GO:0005608 | laminin-3 complex | [LAMA1, LAMB2, LAMC1] |
| GO:0043259 | laminin-10 complex | [LAMA5, LAMB1, LAMC1] |
| GO:0043260 | laminin-11 complex | [LAMA5, LAMB2, LAMC1] |
| GO:0110011 | regulation of basement membrane organization | [LAMA1, LAMB1, LAMB2, LAMC1, NID1, PHLDB2] |

**Table S4. List of up-regulated differentially expressed genes (DEGs)**

| ID | Term |
| --- | --- |
| GO:0005515 | protein binding |
| GO:0071944 | cell periphery |
| GO:0019899 | enzyme binding |
| GO:0033554 | cellular response to stress |
| GO:0043603 | cellular amide metabolic process |
| GO:0005622 | intracellular anatomical structure |
| GO:0044260 | cellular macromolecule metabolic process |
| GO:0031224 | intrinsic component of membrane |
| GO:0016021 | integral component of membrane |
| GO:0006725 | cellular aromatic compound metabolic process |
| GO:0046483 | heterocycle metabolic process |
| GO:0006139 | nucleobase-containing compound metabolic process |
| GO:0070013 | intracellular organelle lumen |
| GO:0090304 | nucleic acid metabolic process |
| GO:0005654 | nucleoplasm |
| GO:0097159 | organic cyclic compound binding |
| GO:1901363 | heterocyclic compound binding |
| GO:0003676 | nucleic acid binding |
| GO:0006725 | cellular aromatic compound metabolic process |
| GO:0034641 | cellular nitrogen compound metabolic process |
| GO:0046483 | heterocycle metabolic process |
| GO:1901360 | organic cyclic compound metabolic process |
| GO:0006139 | nucleobase-containing compound metabolic process |
| GO:0003723 | RNA binding |
| GO:0090304 | nucleic acid metabolic process |
| GO:0006807 | nitrogen compound metabolic process |
| GO:0009058 | biosynthetic process |
| GO:0044237 | cellular metabolic process |
| GO:0044238 | primary metabolic process |
| GO:0071704 | organic substance metabolic process |
| GO:0097159 | organic cyclic compound binding |
| GO:1901363 | heterocyclic compound binding |
| GO:0019222 | regulation of metabolic process |
| GO:0003676 | nucleic acid binding |
| GO:0006725 | cellular aromatic compound metabolic process |
| GO:0009893 | positive regulation of metabolic process |
| GO:0034641 | cellular nitrogen compound metabolic process |
| GO:0043170 | macromolecule metabolic process |
| GO:0044249 | cellular biosynthetic process |
| GO:0046483 | heterocycle metabolic process |
| GO:1901360 | organic cyclic compound metabolic process |
| GO:1901576 | organic substance biosynthetic process |
| GO:0006139 | nucleobase-containing compound metabolic process |
| GO:0009889 | regulation of biosynthetic process |
| GO:0031323 | regulation of cellular metabolic process |
| GO:0051171 | regulation of nitrogen compound metabolic process |
| GO:0060255 | regulation of macromolecule metabolic process |
| GO:0080090 | regulation of primary metabolic process |
| GO:0009059 | macromolecule biosynthetic process |
| GO:0010467 | gene expression |
| GO:0010604 | positive regulation of macromolecule metabolic process |
| GO:0018130 | heterocycle biosynthetic process |
| GO:0019438 | aromatic compound biosynthetic process |
| GO:0031325 | positive regulation of cellular metabolic process |
| GO:0044271 | cellular nitrogen compound biosynthetic process |
| GO:0051173 | positive regulation of nitrogen compound metabolic process |
| GO:1901362 | organic cyclic compound biosynthetic process |
| GO:0010468 | regulation of gene expression |
| GO:0010556 | regulation of macromolecule biosynthetic process |
| GO:0031326 | regulation of cellular biosynthetic process |
| GO:0034654 | nucleobase-containing compound biosynthetic process |
| GO:0090304 | nucleic acid metabolic process |
| GO:0005654 | nucleoplasm |
| GO:0045935 | positive regulation of nucleobase-containing compound metabolic process |
| GO:0051254 | positive regulation of RNA metabolic process |

**Table S5. List of down-regulated differentially expressed genes (DEGs)**

| ID | Term |
| --- | --- |
| GO:0032991 | protein-containing complex |
| GO:0034330 | cell junction organization |
| GO:0046907 | intracellular transport |
| GO:0005788 | endoplasmic reticulum lumen |
| GO:0110165 | cellular anatomical entity |
| GO:1901135 | carbohydrate derivative metabolic process |
| GO:0005829 | cytosol |
| GO:0005975 | carbohydrate metabolic process |
| GO:0006996 | organelle organization |
| GO:0001501 | skeletal system development |
| GO:0009410 | response to xenobiotic stimulus |
| GO:0009605 | response to external stimulus |
| GO:0001503 | ossification |
| GO:0009611 | response to wounding |
| GO:0009986 | cell surface |
| GO:0003824 | catalytic activity |
| GO:0016491 | oxidoreductase activity |
| GO:0003013 | circulatory system process |
| GO:0043232 | intracellular non-membrane-bounded organelle |
| GO:0044260 | cellular macromolecule metabolic process |
| GO:0044282 | small molecule catabolic process |
| GO:0043062 | extracellular structure organization |
| GO:0045229 | external encapsulating structure organization |
| GO:0005102 | signaling receptor binding |
| GO:0048018 | receptor ligand activity |
| GO:0030312 | external encapsulating structure |
| GO:0062023 | collagen-containing extracellular matrix |
| GO:0070013 | intracellular organelle lumen |
| GO:0005654 | nucleoplasm |
| GO:0001655 | urogenital system development |
| GO:0072001 | renal system development |
| GO:0045177 | apical part of cell |
| GO:0098590 | plasma membrane region |
| GO:0007155 | cell adhesion |
| GO:0098609 | cell-cell adhesion |
| GO:0042995 | cell projection |
| GO:0120025 | plasma membrane-bound cell projection |
| GO:1901615 | organic hydroxy compound metabolic process |
| GO:0008202 | steroid metabolic process |
| GO:0012505 | endomembrane system |
| GO:0005783 | endoplasmic reticulum |
| GO:0031226 | intrinsic component of plasma membrane |
| GO:0005887 | integral component of plasma membrane |
| GO:0030054 | cell junction |
| GO:0070161 | anchoring junction |
| GO:0005911 | cell-cell junction |
| GO:0007267 | cell-cell signaling |
| GO:0099177 | regulation of trans-synaptic signaling |
| GO:0099537 | trans-synaptic signaling |
| GO:0097159 | organic cyclic compound binding |
| GO:1901363 | heterocyclic compound binding |
| GO:0003676 | nucleic acid binding |
| GO:0065008 | regulation of biological quality |
| GO:0042592 | homeostatic process |
| GO:0048878 | chemical homeostasis |
| GO:0060322 | head development |
| GO:0007399 | nervous system development |
| GO:0007417 | central nervous system development |
| GO:0007420 | brain development |
| GO:0050793 | regulation of developmental process |
| GO:0051239 | regulation of multicellular organismal process |
| GO:0051240 | positive regulation of multicellular organismal process |
| GO:0051241 | negative regulation of multicellular organismal process |
| GO:2000026 | regulation of multicellular organismal development |
| GO:0005576 | extracellular region |
| GO:0005615 | extracellular space |
| GO:0031982 | vesicle |
| GO:0065010 | extracellular membrane-bounded organelle |
| GO:0070062 | extracellular exosome |
| GO:0016020 | membrane |
| GO:0071944 | cell periphery |
| GO:0031224 | intrinsic component of membrane |
| GO:0005886 | plasma membrane |
| GO:0016021 | integral component of membrane |
| GO:0044281 | small molecule metabolic process |
| GO:0006082 | organic acid metabolic process |
| GO:0006629 | lipid metabolic process |
| GO:0044255 | cellular lipid metabolic process |
| GO:0043436 | oxoacid metabolic process |
| GO:0006631 | fatty acid metabolic process |
| GO:0009889 | regulation of biosynthetic process |
| GO:0009890 | negative regulation of biosynthetic process |
| GO:0010556 | regulation of macromolecule biosynthetic process |
| GO:0031326 | regulation of cellular biosynthetic process |
| GO:0010558 | negative regulation of macromolecule biosynthetic process |
| GO:0031327 | negative regulation of cellular biosynthetic process |
| GO:0051253 | negative regulation of RNA metabolic process |
| GO:0010817 | regulation of hormone levels |
| GO:0051049 | regulation of transport |
| GO:0051051 | negative regulation of transport |
| GO:0046903 | secretion |
| GO:0023061 | signal release |
| GO:0051046 | regulation of secretion |
| GO:1903530 | regulation of secretion by cell |
| GO:0009653 | anatomical structure morphogenesis |
| GO:0009888 | tissue development |
| GO:0048513 | animal organ development |
| GO:0009790 | embryo development |
| GO:0009887 | animal organ morphogenesis |
| GO:0035295 | tube development |
| GO:0048729 | tissue morphogenesis |
| GO:0048598 | embryonic morphogenesis |
| GO:0060429 | epithelium development |
| GO:0048869 | cellular developmental process |
| GO:0009653 | anatomical structure morphogenesis |
| GO:0050793 | regulation of developmental process |
| GO:0051239 | regulation of multicellular organismal process |
| GO:0051094 | positive regulation of developmental process |
| GO:0051240 | positive regulation of multicellular organismal process |
| GO:0022603 | regulation of anatomical structure morphogenesis |
| GO:0045595 | regulation of cell differentiation |
| GO:2000026 | regulation of multicellular organismal development |
| GO:0045597 | positive regulation of cell differentiation |
| GO:1901363 | heterocyclic compound binding |
| GO:0003676 | nucleic acid binding |
| GO:0000785 | chromatin |
| GO:0001067 | transcription regulatory region nucleic acid binding |
| GO:0003677 | DNA binding |
| GO:0010556 | regulation of macromolecule biosynthetic process |
| GO:0019219 | regulation of nucleobase-containing compound metabolic process |
| GO:0034654 | nucleobase-containing compound biosynthetic process |
| GO:0090304 | nucleic acid metabolic process |
| GO:0051252 | regulation of RNA metabolic process |
| GO:0009653 | anatomical structure morphogenesis |
| GO:0048646 | anatomical structure formation involved in morphogenesis |
| GO:0035295 | tube development |
| GO:0035239 | tube morphogenesis |
| GO:0060429 | epithelium development |
| GO:0072359 | circulatory system development |
| GO:0001944 | vasculature development |
| GO:0007507 | heart development |
| GO:0001568 | blood vessel development |
| GO:0048514 | blood vessel morphogenesis |
| GO:0040011 | locomotion |
| GO:0048870 | cell motility |
| GO:0040012 | regulation of locomotion |
| GO:0016477 | cell migration |
| GO:0040017 | positive regulation of locomotion |
| GO:0006935 | chemotaxis |
| GO:2000145 | regulation of cell motility |
| GO:2000147 | positive regulation of cell motility |
| GO:0030334 | regulation of cell migration |
| GO:0030335 | positive regulation of cell migration |
| GO:0048869 | cellular developmental process |
| GO:0009653 | anatomical structure morphogenesis |
| GO:0000902 | cell morphogenesis |
| GO:0032989 | cellular component morphogenesis |
| GO:0048468 | cell development |
| GO:0000904 | cell morphogenesis involved in differentiation |
| GO:0007399 | nervous system development |
| GO:0032990 | cell part morphogenesis |
| GO:0022008 | neurogenesis |
| GO:0030182 | neuron differentiation |
| GO:0048666 | neuron development |
| GO:0005215 | transporter activity |
| GO:0051179 | localization |
| GO:0051234 | establishment of localization |
| GO:0032879 | regulation of localization |
| GO:0006810 | transport |
| GO:0055085 | transmembrane transport |
| GO:0051049 | regulation of transport |
| GO:0051050 | positive regulation of transport |
| GO:0006811 | ion transport |
| GO:0051046 | regulation of secretion |
| GO:1903530 | regulation of secretion by cell |
| GO:0005215 | transporter activity |
| GO:0051234 | establishment of localization |
| GO:0006810 | transport |
| GO:0055085 | transmembrane transport |
| GO:0006811 | ion transport |
| GO:0022857 | transmembrane transporter activity |
| GO:0034220 | ion transmembrane transport |
| GO:0015075 | ion transmembrane transporter activity |
| GO:0015318 | inorganic molecular entity transmembrane transporter activity |
| GO:0022803 | passive transmembrane transporter activity |
| GO:0098655 | cation transmembrane transport |
| GO:0098660 | inorganic ion transmembrane transport |
| GO:0032501 | multicellular organismal process |
| GO:0032502 | developmental process |
| GO:0048856 | anatomical structure development |
| GO:0048869 | cellular developmental process |
| GO:0007275 | multicellular organism development |
| GO:0009653 | anatomical structure morphogenesis |
| GO:0050793 | regulation of developmental process |
| GO:0009888 | tissue development |
| GO:0048513 | animal organ development |
| GO:0048646 | anatomical structure formation involved in morphogenesis |
| GO:0009887 | animal organ morphogenesis |
| GO:0035295 | tube development |
| GO:0045595 | regulation of cell differentiation |
| GO:0048468 | cell development |
| GO:0048731 | system development |
| GO:0007399 | nervous system development |
| GO:0007417 | central nervous system development |
| GO:0022008 | neurogenesis |
| GO:0030182 | neuron differentiation |
| GO:0048666 | neuron development |
| GO:0023052 | signaling |
| GO:0050896 | response to stimulus |
| GO:0007154 | cell communication |
| GO:0009719 | response to endogenous stimulus |
| GO:0042221 | response to chemical |
| GO:0051716 | cellular response to stimulus |
| GO:0023051 | regulation of signaling |
| GO:0010033 | response to organic substance |
| GO:0023056 | positive regulation of signaling |
| GO:0023057 | negative regulation of signaling |
| GO:0070887 | cellular response to chemical stimulus |
| GO:0071495 | cellular response to endogenous stimulus |
| GO:1901700 | response to oxygen-containing compound |
| GO:0009725 | response to hormone |
| GO:0010646 | regulation of cell communication |
| GO:0007166 | cell surface receptor signaling pathway |
| GO:0009966 | regulation of signal transduction |
| GO:0010647 | positive regulation of cell communication |
| GO:0014070 | response to organic cyclic compound |
| GO:0033993 | response to lipid |
| GO:0070848 | response to growth factor |
| GO:0071310 | cellular response to organic substance |
| GO:1901701 | cellular response to oxygen-containing compound |
| GO:0007167 | enzyme-linked receptor protein signaling pathway |
| GO:0006807 | nitrogen compound metabolic process |
| GO:0009058 | biosynthetic process |
| GO:0003676 | nucleic acid binding |
| GO:0006725 | cellular aromatic compound metabolic process |
| GO:0034641 | cellular nitrogen compound metabolic process |
| GO:0043170 | macromolecule metabolic process |
| GO:0044249 | cellular biosynthetic process |
| GO:0046483 | heterocycle metabolic process |
| GO:1901360 | organic cyclic compound metabolic process |
| GO:1901576 | organic substance biosynthetic process |
| GO:0006139 | nucleobase-containing compound metabolic process |
| GO:0009889 | regulation of biosynthetic process |
| GO:0031323 | regulation of cellular metabolic process |
| GO:0051171 | regulation of nitrogen compound metabolic process |
| GO:0060255 | regulation of macromolecule metabolic process |
| GO:0080090 | regulation of primary metabolic process |
| GO:0003677 | DNA binding |
| GO:0009059 | macromolecule biosynthetic process |
| GO:0009890 | negative regulation of biosynthetic process |
| GO:0010467 | gene expression |
| GO:0018130 | heterocycle biosynthetic process |
| GO:0019438 | aromatic compound biosynthetic process |
| GO:0044271 | cellular nitrogen compound biosynthetic process |
| GO:1901362 | organic cyclic compound biosynthetic process |
| GO:0010468 | regulation of gene expression |
| GO:0010556 | regulation of macromolecule biosynthetic process |
| GO:0019219 | regulation of nucleobase-containing compound metabolic process |
| GO:0031326 | regulation of cellular biosynthetic process |
| GO:0034654 | nucleobase-containing compound biosynthetic process |
| GO:0090304 | nucleic acid metabolic process |
| GO:0010558 | negative regulation of macromolecule biosynthetic process |
| GO:0031327 | negative regulation of cellular biosynthetic process |
| GO:0051252 | regulation of RNA metabolic process |

**Table S6. List of UPDOWN-regulated differentially expressed genes (DEGs)**

| ID | Term |
| --- | --- |
| GO:0001503 | ossification |
| GO:0042127 | regulation of cell population proliferation |
| GO:0003013 | circulatory system process |
| GO:0044265 | cellular macromolecule catabolic process |
| GO:0044282 | small molecule catabolic process |
| GO:0007267 | cell-cell signaling |
| GO:0051051 | negative regulation of transport |
| GO:0006520 | cellular amino acid metabolic process |
| GO:0009410 | response to xenobiotic stimulus |
| GO:0010817 | regulation of hormone levels |
| GO:0032989 | cellular component morphogenesis |
| GO:0005975 | carbohydrate metabolic process |
| GO:0044262 | cellular carbohydrate metabolic process |
| GO:0043062 | extracellular structure organization |
| GO:0045229 | external encapsulating structure organization |
| GO:0001655 | urogenital system development |
| GO:0072001 | renal system development |
| GO:0007155 | cell adhesion |
| GO:0098609 | cell-cell adhesion |
| GO:1901615 | organic hydroxy compound metabolic process |
| GO:0008202 | steroid metabolic process |
| GO:0009991 | response to extracellular stimulus |
| GO:0031667 | response to nutrient levels |
| GO:0065008 | regulation of biological quality |
| GO:0042592 | homeostatic process |
| GO:0048878 | chemical homeostasis |
| GO:0051179 | localization |
| GO:0032879 | regulation of localization |
| GO:0051049 | regulation of transport |
| GO:0051050 | positive regulation of transport |
| GO:0055085 | transmembrane transport |
| GO:0006811 | ion transport |
| GO:0022857 | transmembrane transporter activity |
| GO:0034220 | ion transmembrane transport |
| GO:0015075 | ion transmembrane transporter activity |
| GO:0043170 | macromolecule metabolic process |
| GO:0046483 | heterocycle metabolic process |
| GO:0006139 | nucleobase-containing compound metabolic process |
| GO:0010467 | gene expression |
| GO:0090304 | nucleic acid metabolic process |
| GO:0023051 | regulation of signaling |
| GO:0071495 | cellular response to endogenous stimulus |
| GO:0010646 | regulation of cell communication |
| GO:0007166 | cell surface receptor signaling pathway |
| GO:0010647 | positive regulation of cell communication |
| GO:0070848 | response to growth factor |
| GO:0007167 | enzyme-linked receptor protein signaling pathway |
| GO:0009653 | anatomical structure morphogenesis |
| GO:0009888 | tissue development |
| GO:0048513 | animal organ development |
| GO:0009887 | animal organ morphogenesis |
| GO:0035295 | tube development |
| GO:0048729 | tissue morphogenesis |
| GO:0060429 | epithelium development |
| GO:0044281 | small molecule metabolic process |
| GO:0006082 | organic acid metabolic process |
| GO:0006629 | lipid metabolic process |
| GO:0044283 | small molecule biosynthetic process |
| GO:0044255 | cellular lipid metabolic process |
| GO:0016053 | organic acid biosynthetic process |
| GO:0043436 | oxoacid metabolic process |
| GO:0006631 | fatty acid metabolic process |
| GO:0009653 | anatomical structure morphogenesis |
| GO:0050793 | regulation of developmental process |
| GO:0051239 | regulation of multicellular organismal process |
| GO:0051094 | positive regulation of developmental process |
| GO:0051240 | positive regulation of multicellular organismal process |
| GO:0051241 | negative regulation of multicellular organismal process |
| GO:0022603 | regulation of anatomical structure morphogenesis |
| GO:2000026 | regulation of multicellular organismal development |
| GO:0009719 | response to endogenous stimulus |
| GO:0042221 | response to chemical |
| GO:0051716 | cellular response to stimulus |
| GO:0010033 | response to organic substance |
| GO:0070887 | cellular response to chemical stimulus |
| GO:0071495 | cellular response to endogenous stimulus |
| GO:1901700 | response to oxygen-containing compound |
| GO:0071310 | cellular response to organic substance |
| GO:1901701 | cellular response to oxygen-containing compound |
| GO:0009653 | anatomical structure morphogenesis |
| GO:0048646 | anatomical structure formation involved in morphogenesis |
| GO:0035295 | tube development |
| GO:0035239 | tube morphogenesis |
| GO:0060429 | epithelium development |
| GO:0072359 | circulatory system development |
| GO:0001944 | vasculature development |
| GO:0001568 | blood vessel development |
| GO:0001525 | angiogenesis |
| GO:0048514 | blood vessel morphogenesis |
| GO:0032501 | multicellular organismal process |
| GO:0032502 | developmental process |
| GO:0048856 | anatomical structure development |
| GO:0007275 | multicellular organism development |
| GO:0009653 | anatomical structure morphogenesis |
| GO:0050793 | regulation of developmental process |
| GO:0009888 | tissue development |
| GO:0048513 | animal organ development |
| GO:0048646 | anatomical structure formation involved in morphogenesis |
| GO:0009887 | animal organ morphogenesis |
| GO:0048731 | system development |
| GO:0007399 | nervous system development |
| GO:0009719 | response to endogenous stimulus |
| GO:0042221 | response to chemical |
| GO:0010033 | response to organic substance |
| GO:0070887 | cellular response to chemical stimulus |
| GO:0071495 | cellular response to endogenous stimulus |
| GO:1901698 | response to nitrogen compound |
| GO:1901700 | response to oxygen-containing compound |
| GO:0009725 | response to hormone |
| GO:0010243 | response to organonitrogen compound |
| GO:0014070 | response to organic cyclic compound |
| GO:0033993 | response to lipid |
| GO:0070848 | response to growth factor |
| GO:0071310 | cellular response to organic substance |
| GO:1901699 | cellular response to nitrogen compound |
| GO:1901701 | cellular response to oxygen-containing compound |
| GO:0071417 | cellular response to organonitrogen compound |
| GO:0007167 | enzyme-linked receptor protein signaling pathway |
