# Supplementary material for: A human liver organoid platform for hepatotoxicity assessment: evaluation using reference compounds
Source: Front Toxicol. 2026 Jun 15;8:1805474. doi: 10.3389/ftox.2026.1805474 (PMC13310563; doi:10.3389/ftox.2026.1805474)
Supplement: Supplementary file 2 [file DataSheet1.docx]

_
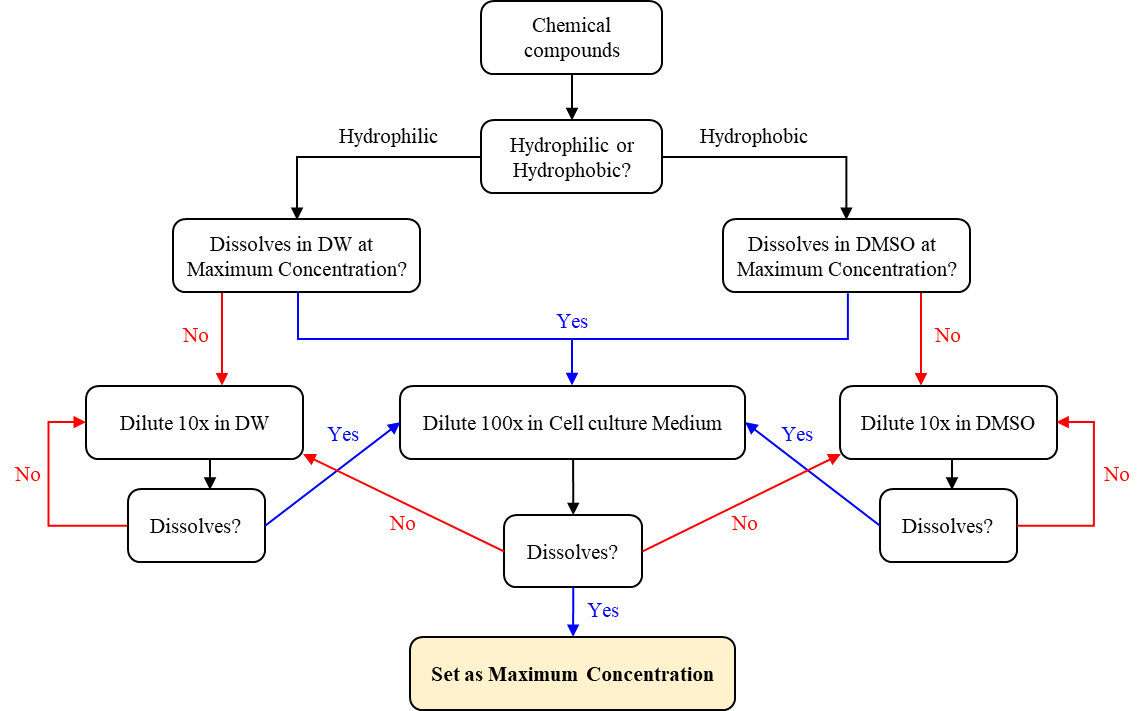


**Figure S1. Flowchart of solubility testing for ten chemical compounds.**

The solubility tests for the chemical compounds were conducted by dissolving hydrophilic compounds in DW and hydrophobic compounds in DMSO. If the maximum solubility exceeded 1M, a test was performed at 1M to ensure that the stock concentration did not exceed 1M. Compounds that dissolved well in the solvent were further tested in the cell culture medium, as previously described.

**
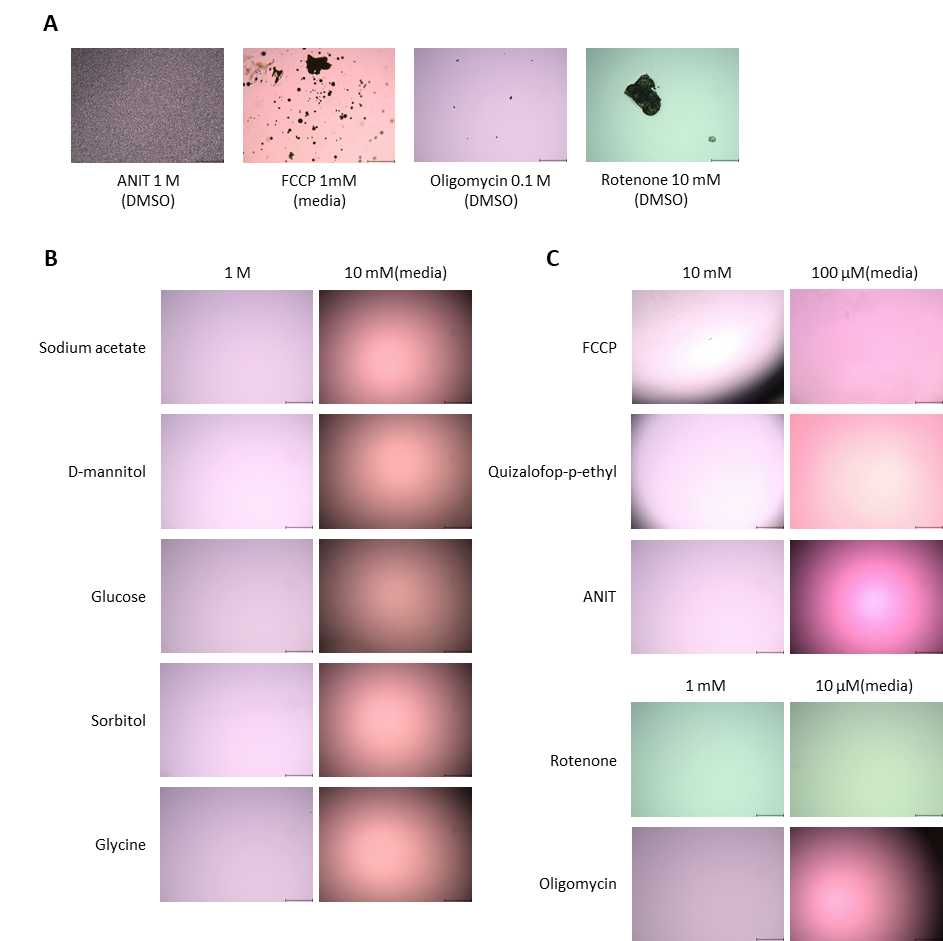
**

**Figure S2. Microscopic images from solubility tests of ten chemical compounds.**

Images of compounds dissolved in solvent and cell culture medium. (A) Crystals formed from each compound at high concentrations. (B) Hydrophilic and (C) hydrophobic compounds at their highest soluble concentration. All images were captured at 40× magnification with a scale bar of 500 μm.

**
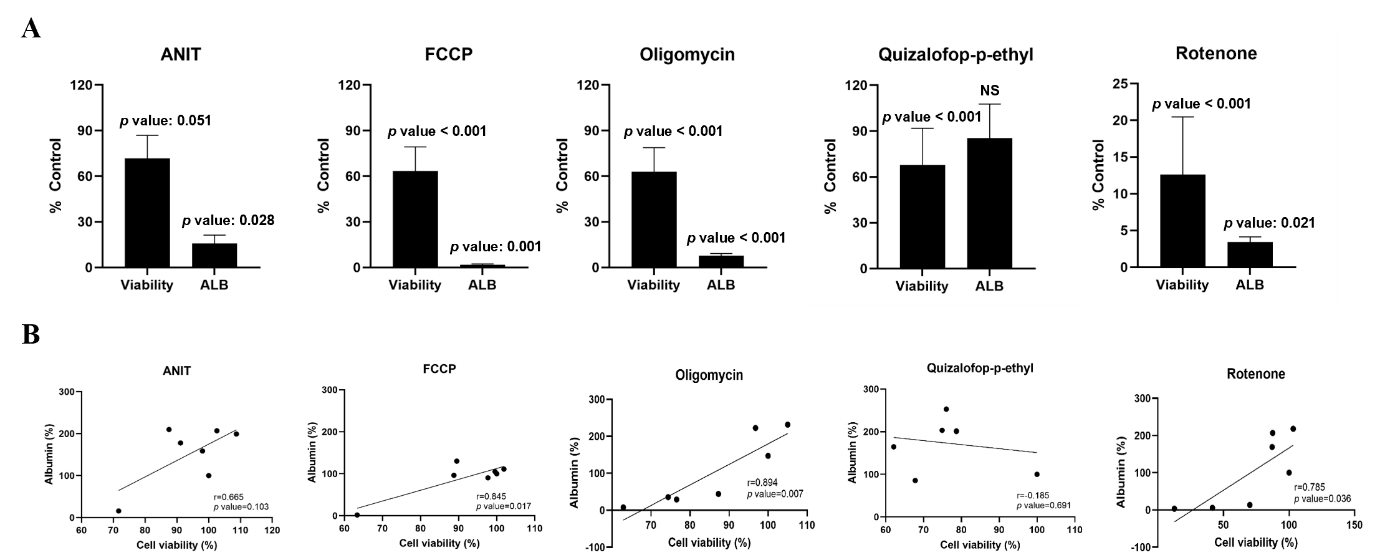
**

**Figure S3. Comparison of sensitivity to toxicity and correlation analysis between cell viability and albumin secretion.**

Additional analyses were performed using cell viability and albumin secretion values measured on day 6 post-treatment in the liver organoids. (A) Relative reduction in cell viability and albumin secretion at the highest concentration of each of the five toxic compounds compared with the control group. Statistical significance was determined using Student’s *t*-test, with *p* values indicated. (B) Correlation between cell viability and albumin secretion was assessed using Pearson’s correlation analysis.


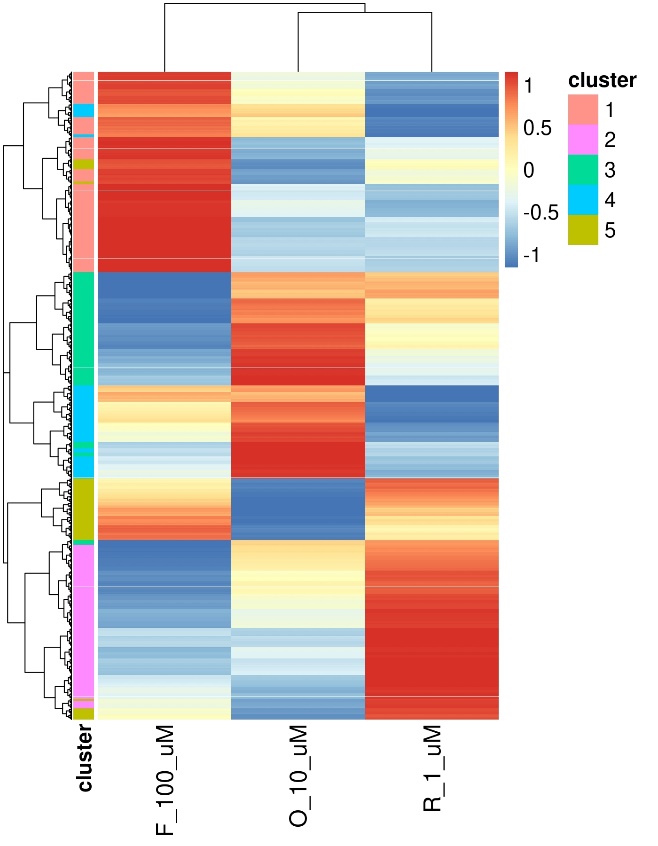


**Figure S4. K-means clustering heatmaps of differentially expressed genes.**

Heatmaps showing five gene expression clusters generated by k-means clustering (*k* = 5). These visualizations summarize the transcriptional patterns across treatment groups. R, rotenone; F, FCCP; O, oligomycin.


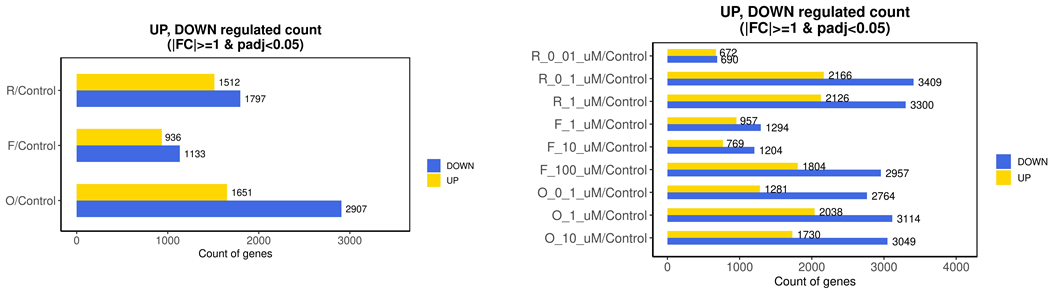


**Figure S5. Differentially expressed gene (DEG) counts across treatments and concentrations.** Number of up- and down-regulated genes (|FC| ≥ 1 and padj < 0). R, rotenone; F, FCCP; O, oligomycin.
